# Predictive neural processing of self-generated hand and tool actions in patients with schizophrenia spectrum disorders and healthy individuals

**DOI:** 10.1038/s41398-025-03306-6

**Published:** 2025-03-17

**Authors:** Christina V. Schmitter, Mareike Pazen, Lukas Uhlmann, Bianca M. van Kemenade, Tilo Kircher, Benjamin Straube

**Affiliations:** 1 Department of Psychiatry and Psychotherapy, University of Marburg, Rudolf-Bultmann-Strasse 8, Marburg, Germany; 2 Center for Mind, Brain and Behavior (CMBB), University of Marburg and Justus Liebig University Giessen, Hans-Meerwein-Strasse 6, Marburg, Germany; 3 Center for Psychiatry, Justus Liebig University Giessen, Klinikstrasse 36, Giessen, Germany

**Keywords:** Neuroscience, Schizophrenia

## Abstract

Schizophrenia spectrum disorders (SSD) have been linked to dysfunctions in the predictive neural suppression of sensory input elicited by one’s own actions. Such motor predictions become particularly challenging during tool use and when feedback from multiple sensory modalities is present. In this study, we investigated the neural correlates and potential dysfunctions of action feedback processing in SSD during tool use actions and bimodal sensory feedback presentation. Patients with SSD (N_Total_ = 42; schizophrenia N_F20_ = 34; schizoaffective disorder N_F25_ = 6; other N = 2) and healthy controls (HC, N = 27) performed active or passive hand movements with or without a tool and received unimodal (visual; a video of their hand movement) or bimodal (visual and auditory) feedback with various delays (0, 83, 167, 250, 333, 417 ms). Subjects reported whether they detected a delay. A subgroup (N_SSD_ = 20; N_HC_ = 20) participated in an identical fMRI experiment. Both groups reported fewer delays in active than passive conditions and exhibited neural suppression in all conditions in occipital and temporoparietal regions, cerebellum, and SMA. Group differences emerged in right cuneus, calcarine, and middle occipital gyrus, with reduced active-passive differences in patients during tool use actions and in bimodal trials during actions performed without a tool. These results demonstrate for the first time that, although patients and HC show similarities in neural suppression, higher-level visual processing areas fail to adequately distinguish between self- and externally generated sensory input in patients, particularly in complex action feedback scenarios involving bimodal action feedback and feedback elicited by tool use actions.

## Introduction

Hallucinations and ego-disturbances are core symptoms of schizophrenia spectrum disorders (SSD), characterized by misperceiving one’s own actions and thoughts as externally produced or controlled [1]. Their emergence has been linked to dysfunctions of internal forward models that predict the sensory consequences of our own movements using a copy of the motor command (efference copy). If the prediction matches the actual sensory input, the perception of incoming sensory information is suppressed, informing the system that the sensory input was self-generated [2–4]. Reduced suppression of self-generated sensory input in SSD, both on the behavioral [5, 6] and neural level [6–8], suggests disruptions of this predictive mechanism that contribute to source misattributions and consequently increase the risk for the emergence of hallucinations and ego-disturbances [9–11].

In healthy individuals, neural suppression for active movements was demonstrated in brain regions for early sensory processing. However, activity modulations during the processing of self-generated action feedback have been linked to widespread networks including, for instance, premotor regions and the supplementary motor area (SMA) which were associated with the generation of the efference copy signal used for prediction generation [3, 12]. Posterior parietal regions, including the angular gyrus, were found to compare predicted and perceived sensory input, thereby contributing to the subjective feeling of agency [13, 14]. Finally, the cerebellum has been suggested as the location of internal forward models and is therefore strongly associated with action feedback prediction [4, 15–17]. In patients with SSD, reduced neural suppression was demonstrated in visual processing areas (including cuneus and middle occipital gyrus) [6], as well as in areas for auditory [18], and somatosensory processing [8]. Patients also showed reduced responses of the angular gyrus to violations of the feeling of agency [19, 20] and a different pattern of cerebellar activity during voluntary movements [21]. These findings indicate widespread deficits in action monitoring and the neural processing of sensory input elicited by one’s own actions.

Importantly, in our everyday lives, we often apply tools to interact with other objects in the environment [22, 23]. These tools are not part of our bodies, yet we are still able to perceive the sensory consequences of tool use actions as self-generated [24]. According to behavioral findings in healthy individuals, the predictive processing of natural limb movements and tool use actions show a substantial mechanistic overlap [25]. A neuroimaging study further found tool use actions and actions directly performed with the own hand to share a large pattern of BOLD suppression, indicating that sensory consequences of tool use actions are similarly predictable [26]. Nonetheless, tool use actions pose an additional challenge to these predictive mechanisms, as the tool must be considered by the internal forward model and integrated into the prediction generation process. Thus, additional resources are required in sensorimotor areas for incorporating the tool into the prediction model in order to achieve comparable prediction performance as for actions executed without tools [26]. Patients with SSD were shown to exhibit general impairments in tool use performance [27, 28], and this impairment was associated with reduced gray matter volume in areas related to action monitoring, including parietal and premotor areas [27]. It may therefore be assumed that these general deficits in tool use, along with the specific challenges these actions pose to predictive mechanisms based on the forward model, result in particularly poor predictions of the sensory consequences elicited by actions performed with the aid of a tool. The deficit in attributing self-generated sensory input to one’s own action could consequently be particularly noticeable in situations where tools are frequently used. However, evidence for this claim is thus far missing.

Furthermore, the sensory consequences of our actions typically manifest in multiple sensory modalities. In healthy individuals, multimodal action feedback is associated with the concurrent suppression of neural activity in multiple sensory processing regions [4]. In patients with SSD, dysfunctions in the integration of information from multimodal stimuli have been frequently demonstrated, and these have been linked to dysfunctional processes in multisensory integration areas such as the posterior superior temporal sulcus and to disturbed fronto-temporal connectivity [29, 30]. Therefore, it is conceivable that predicting action feedback, which is concurrently available from multiple sensory modalities, is particularly challenging for patients when the multimodal feedback cannot be adequately integrated and attributed to a common cause, i.e., one's own movement. However, whether and how the prediction deficit in patients with SSD manifests in a multimodal action feedback context has not yet been investigated.

To address these knowledge gaps, the aim of the present study was to investigate the predictive processing of tool use actions and multimodal action feedback in SSD. During fMRI, patients and healthy controls (HC) performed active and passive wrist movements with or without a tool and received unimodal (visual) or bimodal (visual and auditory) feedback at various delays. Subjects reported whether they detected any feedback delay. At the behavioral level, reduced perceptual sensitivity, i.e., suppression of self-generated action feedback, was expected to manifest in terms of reduced detection performance in active compared to passive conditions. Patients were expected to show reduced active-passive differences in delay detection and neural activations indicating dysfunctions in action feedback processing. Furthermore, we expected that the deficits in patients would manifest particularly severely for the processing of tool use actions and bimodal action feedback due to the additional challenges these factors may pose to predictive mechanisms. This may be reflected in activity differences between patients and HC in these conditions in primary sensory, motor, and sensorimotor areas, such as the cerebellum.

## Materials and methods

### Participants

Forty-two subjects with SSD and 27 HC matched for age, sex, and education participated in the study and provided informed consent (see Table 1). SSD patients had an ICD-10 diagnosis of schizophrenia (N_F20_ = 34) or schizoaffective disorder (N_F25_ = 6). One patient was diagnosed with acute polymorph psychotic disorder (F23), and one patient had drug-induced psychosis (F19.7). All subjects were right-handed and reported (corrected-to-) normal vision and hearing. HC subjects did not report any history of psychiatric disorders or any immediate relatives with diagnosed SSD (for further details on exclusion criteria see S1 in the supplementary material). Twenty patients and 20 HC completed the fMRI experiment. The other 22 patients and 7 HC participated in a behavioral experiment outside the MRI scanner. Ethics approval (reference number: 99/15) was granted by the ethics committee of the medical faculty of the Philipps-University Marburg, Germany, in accordance with the Declaration of Helsinki.

**Table 1:** Group characteristics.

	SSD (N = 42)	HC (N = 27)	Group comparisons		
			*t*-value	*p*-value	*d*
***Demographics***					
Sex					
Male	32 (76.19%)	21 (77.78%)			
Female	10 (23.81%)	6 (22.22%)			
Age (in years)	38.40 ± 9.79 (21–56)	38.41 ± 10.69 (20–60)	0.001	0.999	<0.001
Education					
Lower secondary	21 (50%)	13 (48.15%)			
Upper secondary	17 (40.48%)	9 (33.33%)			
Tertiary	4 (9.52%)	5 (18.52%)			
***Handedness***					
Laterality quotient (EHI)	87.46 ± 17.08 (38.46–100)	84.31 ± 26.27 (20–100)	−0.601	0.550	−0.148
***Clinical measures***					
Positive symptoms (SAPS total score)	**11.90** ± **10.83** (**0–40)**^**a**^	**1.52** ± **2.12** (**0–9)**	**−4.908**	**<0.001**	**−1.216**
Negative symptoms (SANS total score)	**13.68** ± **14.06** (**0–52)**^**a**^	**1.15** ± **1.81** (**0–8)**	**−4.595**	**<0.001**	**−1.139**
***Neuropsychological control measures***					
Attention (d2)					
Number of items worked on	**392.00** ± **106.41** (**211–586)**^**a**^	**458.26** ± **91.68** (**317–639)**	**2.650**	**0.010**	**0.657**
Total error score	18.78 ± 15.39 (1–72)^a^	21.85 ± 14.35 (3–57)	0.827	0.411	0.205
Executive functions					
TMT A (in s)	33.17 ± 11.39 (17–71)	31.63 ± 16.04 (16–84)	−0.465	0.643	−0.115
TMT B (in s)	**83.07** ± **33.83** (**28–167)**	**62.11** ± **25.62** (**27–157)**	**−2.741**	**0.008**	**−0.679**
Intelligence (MWT-B-IQ)	101.68 ± 13.11 (78–143)^a^	106.07 ± 14.14 (85–136)	1.310	0.195	0.325
Short term memory (WAIS-DS)					
Forward	9.55 ± 1.85 (6–15)	9.63 ± 1.64 (7–13)	0.187	0.852	0.046
Backward	7.83 ± 1.86 (2–11)	8.33 ± 1.64 (5–12)	1.140	0.258	0.281
***Antipsychotic medication***					
None	4	27			
First generation	7^b^	0			
Second generation	37^b^	0			

^a^N = 41.

^b^Six patients were treated with both first and second generation antipsychotics.

*d2* d2 Test of Attention, *EHI* Edinburgh Handedness Inventory, *MWT-B* Multiple choice vocabulary test, *SANS* Scale for the Assessment of Negative Symptoms, *SAPS* Sale for the Assessment of Positive Symptoms, *TMT* Trail Making Test, *WAIS-DS* Wechsler Adult Intelligence Scale - Digit Span. Data for continuous variables are reported as mean ± standard deviation (range). For categorical data, the percentage of cases in each category is shown in brackets. Potential differences between groups in age, handedness, clinical scores, and neuropsychological control measures were assessed by independent-samples *t*-tests. The corresponding *t-*values, *p*-values and Cohen’s *d* are provided for each test. Significant group differences are presented with bold values (*p* < 0.05, uncorrected). There were no significant age differences between the groups, and both groups had similar sex and education ratios, indicating that they were comparable regarding basic demographic variables. Similar comparisons were performed on the fMRI samples only (20 subjects per group) and are provided in the supplementary material S2.

### Experimental design and procedure

Subjects performed the experiment either during fMRI data acquisition while lying in the MRI scanner, or alternatively, if MRI was contraindicated, during a behavioral session outside the scanner while sitting at a desk in an upright position. Subjects held onto the handle of a custom-made MR-compatible device with the right hand, which could be moved along a circular arc (range: ~5 cm, angle: 27°) from a starting position on the left to a turning point on the right (and back). The movement had to be performed either actively by the subjects themselves, or it was induced passively with compressed air. Since both active and passive movements were associated with similar tactile and proprioceptive sensations, this manipulation allowed for the isolation of forward model-based predictive mechanisms, which should only be present during actively executed movements. To study movements performed without a tool, subjects grabbed the handle of the device with their own hand for half of the trials. To study movements performed with a tool, for the remaining trials, a custom-made tool was mounted onto the handle. Subjects held onto the tool with a whole hand grip and performed the same hand movement to move the handle (for details see Supplementary Fig. 1). In all conditions subjects were provided with video feedback of the movements. For this purpose, during the hand conditions, the movement of the hand, and during the tool conditions, the movement of the tool (while the subject’s hand remained out of view) were recorded in real-time using a high-speed camera (~4 ms refresh rate, MRC High Speed, MRC Systems GmbH, Heidelberg, Germany). Subjects saw the video on a monitor (refresh rate 60 Hz) which was located behind the MRI scanner and could be viewed via a mirror mounted onto the head coil. Furthermore, the effect of bimodal action feedback was assessed by coupling the visual feedback with the sound of the movement device when hitting the turning and starting point in half of the trials. Due to the scanner noise, this sound was prerecorded and delivered through MR-compatible headphones (MR-Confon Optimel, Magdeburg, Germany). In each trial, the sensory feedback was displayed with one of six different levels of delay relative to the movement (0, 83, 167, 250, 333, or 417 ms, in addition to the inherent setup delay of 43 ms). In bimodal trials, the visual and auditory feedback delay was always the same. Subjects reported via keypress after each trial whether they detected a delay. Together, this manipulation resulted in a mixed experimental design with the between-subjects factor Group (HC vs. SSD) and the within-subjects factors Movement type (active vs. passive), Instrumentality (hand vs. tool), and Modality (unimodal vs. bimodal). The experiment was divided into four runs with 48 trials each (resulting in 8 trials for each of the four experimental conditions and each delay level). Subjects were familiarized with all procedures in a separate training session (see S4 in the supplementary material for further details on the experimental procedure and training session).

### fMRI data acquisition

MRI data were collected using a 3T MR Magnetom Trio Tim scanner (Siemens, Erlangen, Germany) at the Department of Psychiatry and Psychotherapy, Marburg, Germany, using a 12-channel head coil (see [26] where the same data acquisition procedure has previously been applied in healthy subjects). Functional data were acquired using a T2*-weighted gradient echo echoplanar imaging sequence (repetition time [TR]: 1650 ms; echo time [TE]: 25 ms; flip angle: 70°). For each experimental run, 330 volumes were obtained, each containing 34 transversal slices acquired parallel to the intercommissural line (a plane through the anterior and posterior commissure) in descending order (64 × 64 matrix, field of view [FoV]: 192 × 192 mm, slice thickness: 4 mm, voxel size: 3 × 3 × 4.6 mm [including 15% gap]), allowing each volume to cover the whole brain (incl. cerebellum). Anatomical images were obtained using a T1 weighed magnetization prepared rapid gradient echo (MPRAGE) sequence (176 slices ascending, TR: 1900 ms, TE: 2.26 ms, flip angle: 9°, 256 × 256 matrix, FoV: 256 × 256 mm, slice thickness: 1 mm, voxel size: 1 × 1 × 1.5 mm [including 50% gap]). To minimize head motion subjects’ heads were stabilized using foam pads.

### Data analyses

#### Behavioral data

We conducted a sanity check to determine whether subjects were able to perform the delay detection task adequately (for details see S5 in the supplementary material). Trials in which no response was given were excluded from the analysis of behavioral data (SSD: 1.24%, HC: 0.64% of all trials). Psychometric functions, modeled as cumulative Gaussian distribution functions, were fit to these data using version 4 of the Psignifit toolbox [31] for Python version 3.11 (Python Software Foundation, https://www.python.org/). Delay detection thresholds (i.e., the delay detected in 50% of trials) and the functions’ width (i.e., the increase in the amount of delay between 5% and 95% detection performance) were extracted from the psychometric functions. The detection thresholds served as a measure of overall delay detection performance, with lower values indicating better performance. The functions’ widths reflected the rate of change in detected delays as delay levels increased, representing the ability to discriminate between different delays. Two 4-way mixed ANOVAs with the between-subjects factor Group (HC vs. SSD) and the within-subjects factors Instrumentality (hand vs. tool), Movement type (active vs. passive), and Modality (unimodal vs. bimodal) were then performed on both performance measures. Furthermore, for all effects, Bayes factors were calculated based on Bayesian ANOVAs performed with default priors. They are reported as the inclusion of a particular effect (BF_incl_), determined by the ratio of the likelihood of the data under the model containing the effect to the likelihood under the next simpler model without the effect [32]. All statistics were performed with JASP (Version 0.14.1) [33]. We were primarily interested in the two-way interaction Group x Movement type to assess differences in behavioral suppression effects between patients and HC. Furthermore, we were interested in the four-way interaction Group x Movement type x Instrumentality x Modality to examine whether differences in predictive mechanisms of the forward model between the groups manifest specifically for actions performed with tools and trials with bimodal action feedback.

#### MRI data

MRI data were analyzed using Statistical Parametric Mapping (SPM12; www.fil.ion.ucl.ac.uk) in MATLAB (Version 2017a, Mathworks, Sherborn, Massachusetts). As in a previous study with a similar experimental paradigm [26], for data preprocessing, standard realignment, coregistration between structural and functional scans, segmentation, normalization (Montreal Neurological Institute [MNI] template, resampled to 2 × 2 × 2 mm voxels), and smoothing (8 mm full-width at half maximum Gaussian kernel) functions of SPM12 were applied.

To obtain an objective measure on which data to exclude due to excessive head motion, we additionally calculated framewise displacement, i.e., subjects’ head movement from one volume to the next using the motion outlier tool for the FMRIB Software Library (FSL) [34]. Any run in which more than 10% of framewise displacement values [35] were labelled as outliers at a threshold of 0.75 mm were excluded. A total of 5 runs were excluded across all subjects based on this criterion. One additional run had to be excluded due to technical issues during data collection.

Preprocessed data were analyzed using a GLM. Regressors of interest were defined for the period between camera onset and camera offset (4000 ms) for each within-subject condition separately. The period for the cue ‘Ready’ (1500 ms) and the period during which the question ‘Delay’ was displayed (from its onset until the response was given) were included as regressors of no interest. Regressors were convolved with the canonical hemodynamic response function (HRF). Furthermore, the first temporal derivative (td) of each regressor was included to account for temporal shifts in the BOLD signal. Realignment parameters were included to account for head motion. Low frequencies were removed using a high-pass filter with a cut-off period of 128 s. Individual parameter estimates (betas) and *t*-statistic images were calculated (based on the HRF) for each condition contrasted against an implicit baseline, which comprised all events that were not captured by any of the regressors (e.g., the inter-trial interval). This resulted in 2 × 2 × 2 contrasts on single subject level: hand active unimodal (HandActUni), hand active bimodal (HandActBi), hand passive unimodal (HandPasUni), hand passive bimodal (HandPasBi), tool active unimodal (ToolActUni), tool active bimodal (ToolActBi), tool passive unimodal (ToolPasUni), tool passive bimodal (ToolPasBi). Contrast estimates of these contrasts were then entered into a full factorial group analysis. Automated Anatomical Labelling 3 (AAL3) [36] was used to label significant activations based on peak activation voxels. The statistical threshold for whole-brain analyses was determined by Monte Carlo simulations with 10.000 iterations [37] using the estimated smoothness of our data (13.5 mm). Simulations suggested that a minimum of 94 continuous voxels activated at *p* < 0.005 uncorrected is sufficient to correct for multiple comparisons at cluster level (*p* < 0.05). We only report clusters activated at the mentioned threshold. For all fMRI analyses, we used an inclusive mask of grey and white matter, which was created from each subject’s structural scan and then averaged across the whole sample.

We used *t*-contrasts to examine our hypotheses. To compare HC and SSD with regard to sensory suppression, we investigated similarities using a conjunction analysis [38] of the Passive > Active contrast for both groups and separately for each of the four remaining experimental conditions (HandUni, HandBi, ToolUni, ToolBi). We further assessed with two-way interaction contrasts between the factors Group and Movement type whether neural processing of actively vs. passively generated sensory input differed between SSD and HC depending on the experimental manipulations. Furthermore, we investigated whether group differences in neural processing were modulated by the instrument used for performing the action (hand vs. tool) and the feedback modality (unimodal vs. bimodal) by calculating four-way interaction contrasts including all four factors.

Because of occasional problems with the algorithm of the movement device or because subjects did not perform the movements correctly (i.e., it did not precisely reach the turning or end point), in some bimodal trials the presentation of the sound may not have been triggered. Therefore, we additionally performed the behavioral and fMRI analyses described above while excluding all trials with errors in movement execution to make sure that these issues did not affect the reported results (see S7 in the supplementary material). Finally, for patients, we performed exploratory correlation analyses between the main behavioral and neural outcomes and their current level of ego-disturbances and hallucinations as well as the chlorpromazine (CPZ) equivalents of their antipsychotic medication at the time of the study. These assessments aimed to determine whether the observed results were influenced by the medication or current symptom severity, respectively (results of these analyses are reported in S8 and S9 in the supplementary material).

## Results

### Behavioral results

**Fig. 1:**
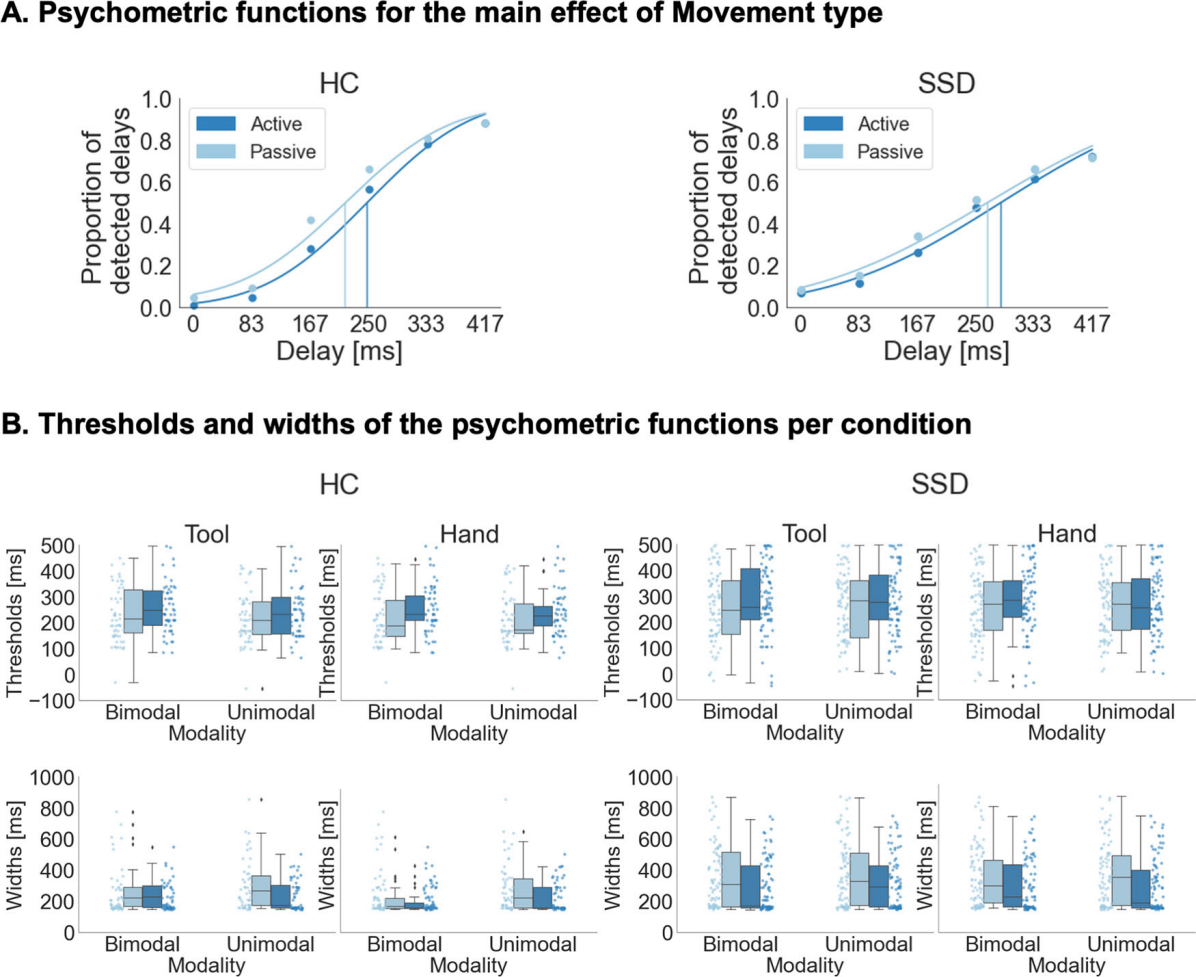
Behavioral results. **A**: Psychometric functions were fitted to the delay detection data for each individual experimental condition and are displayed here for illustration purposes for active and passive conditions and separately for both groups. **B**. Thresholds and widths of the psychometric functions are displayed for each individual condition. Thresholds were larger, and thus detection performance was significantly worse in active than in passive conditions. Furthermore, the functions were significantly wider in active than in passive conditions and for patients with SSD than for HC. N_SSD_ = 42, N_HC_ = 27.

Behavioral results are based on the full sample (N_SSD_ = 42; N_HC_ = 27) and are displayed in Fig. 1. The mixed ANOVA of delay detection thresholds revealed a significant main effect of Movement type [*F*(1, 67) = 15.994, *p* < 0.001, η_p_^2^ = 0.193, BF_incl_ = 1227.016] indicating larger thresholds and thus fewer detected delays for active (*M* = 268.445, *SE* = 13.209) compared to passive (*M* = 246.015, *SE* = 13.421) actions, and very strong evidence for this effect according to the inclusion Bayes factor. The main effects of Instrumentality [*F*(1, 67) = 2.009, *p* = 0.161, η_p_^2^ = 0.029, BF_incl_ = 0.115] and Modality [*F*(1, 67) = 3.018, *p* = 0.087, η_p_^2^ = 0.043, BF_incl_ = 0.058] and the interactions of the experimental factors did not reach significance (all *p* > 0.068, all BF_incl_ < 0.1). Furthermore, the main effect of Group [*F*(1, 67) = 2.023, *p* = 0.160, η_p_^2^ = 0.029, BF_incl_ = 0.152] and the interactions of the experimental factors with the Group factor were not significant (all *p* > 0.206, all BF_incl_ < 0.141). See S6 in the supplementary material for a detailed summary of all effects and Bayes factors.

The mixed ANOVA of the widths of the psychometric functions revealed a significant main effect of Group [*F*(1, 67) = 11.234, *p* = 0.001, η_p_^2^ = 0.144, BF_incl_ = 3.860] indicating wider curves and thus a reduced ability to discriminate between delay levels in patients with SSD (*M* = 332.566, *SE* = 18.566) than in HC (*M* = 246.675, *SE* = 13.584), and moderate evidence for this effect, according to the inclusion Bayes factor. Additionally, this analysis revealed a significant main effect of Movement type [*F*(1, 67) = 13.781, *p* = < 0.001, η_p_^2^ = 0.171, BF_incl_ = 765.365] with very strong evidence for this effect, according to the inclusion Bayes factor, and wider functions for passive (*M* = 327.192, *SE* = 17.113) compared to active (*M* = 270.720, *SE* = 12.992) actions. The main effects of Instrumentality [*F*(1, 67) = 2.804, *p* = 0.099, η_p_^2^ = 0.040, BF_incl_ = 0.064] and Modality [*F*(1, 67) = 2.825, *p* = 0.097, η_p_^2^ = 0.040, BF_incl_ = 0.096], and the interactions of the experimental factors did not reach significance (all *p* > 0.236, all BF_incl_ < 0.075). Furthermore, the interactions of the experimental factors with the Group factor were not significant (all *p* > 0.210, all BF_incl_ < 0.167).

### fMRI results

#### Group similarities

**Fig. 2:**
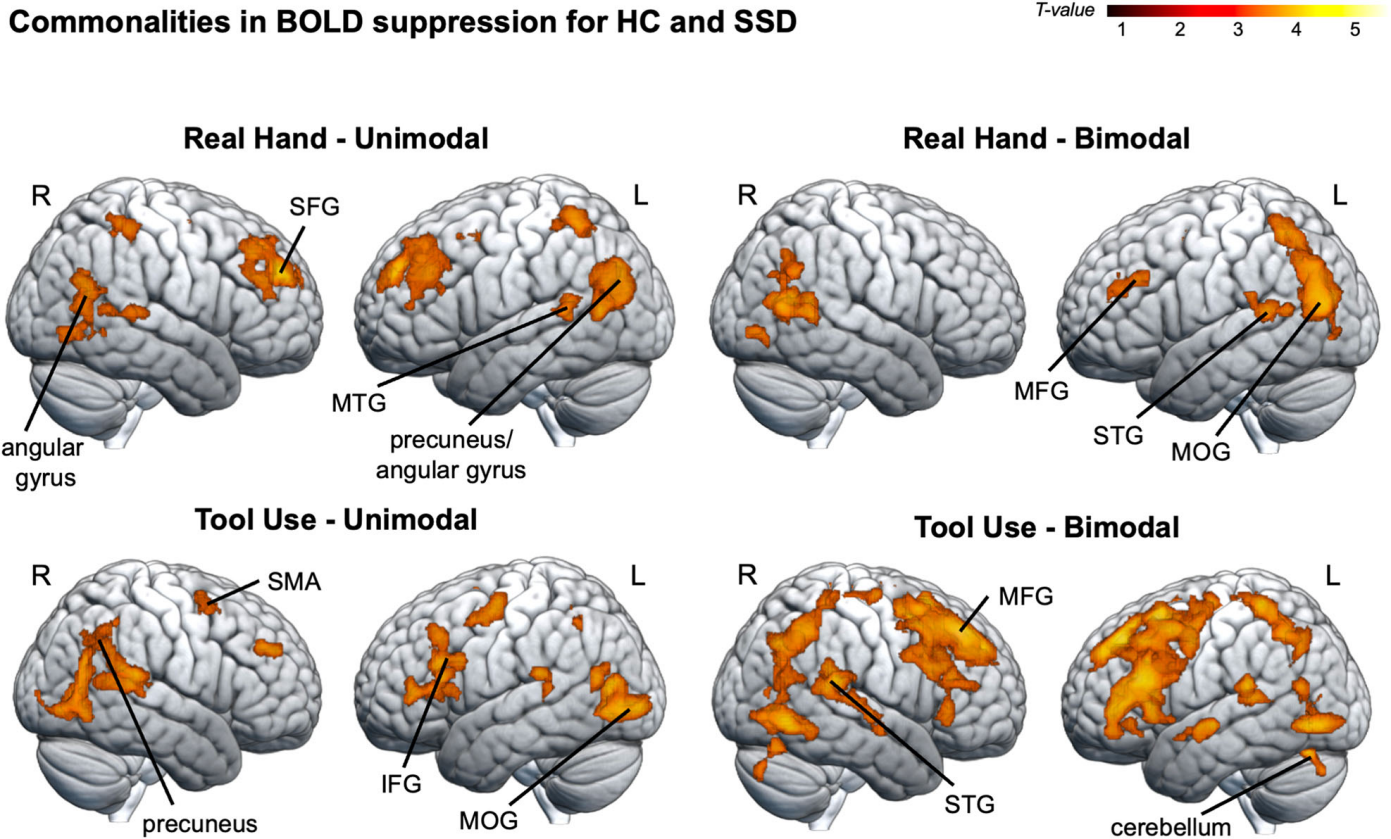
Commonalities in BOLD suppression for SSD and HC. Commonalities in BOLD suppression (i.e., less BOLD signal in active compared to passive conditions) for HC and SSD were assessed by means of conjunction analyses for four experimental conditions (HandUni, HandBi, ToolUni, ToolBi). A common pattern of BOLD suppression emerged for both groups in a range of parietal (e.g., precuneus), temporal (e.g., middle and superior temporal gyri, fusiform gyrus), and frontal regions (e.g., middle and superior frontal gyri). Significance threshold: *p* < 0.005 (unc.), with a cluster extent threshold of 94 voxels, *p* < 0.05 Monte Carlo cluster level corrected [37]. *MFG* middle frontal gyrus, *MTG* middle temporal gyrus, *SFG* superior frontal gyrus, R right, L left. N_SSD_ = 20, N_HC_ = 20.

The conjunction analyses of the Passive > Active contrast for HC and SSD, performed for real hand and tool use as well as for unimodal and bimodal conditions (HandUni, HandBi, ToolUni, ToolBi) each revealed clusters of activation with a similar pattern in a range of visual processing regions, including the middle occipital gyrus and the fusiform gyrus, as well as preparatory motor areas, including the SMA and the cerebellum. Activations also extended to further parietal (e.g., precuneus), temporal (e.g., middle and superior temporal gyri) and frontal regions (e.g., middle and superior frontal gyri). This indicates that actions performed with the hand and with the tool, as well as unimodal and bimodal action feedback were associated with a similar pattern of BOLD suppression in these regions in both patients and HC (see Fig. 2 and Table 2).

**Table 2:** Anatomical locations for conjunction analysis (Passive > Active).

Anatomical Locations (Local Maxima)	Hemisphere	x	y	z	T	No. Voxels
*Commonalities in BOLD suppression for HC and SSD during real hand and unimodal trials*						
Superior medial frontal gyrus	R	6	50	30	4.03	1722
Middle frontal gyrus	L	−30	34	28	3.32	
Superior frontal gyrus	R	22	32	42	3.27	
Precuneus	L	−6	−60	16	3.85	2961
Calcarine	L	−14	−64	14	3.72	
Angular gyrus	L	−44	−72	30	3.67	
Angular gyrus	R	42	−62	24	3.52	383
Middle temporal gyrus	R	44	−64	14	3.15	
Middle temporal gyrus	R	52	−66	16	2.87	
Supplementary motor area	L	−8	4	46	3.31	230
Supplementary motor area	R	2	−6	52	3.06	
Supplementary motor area	R	4	−18	52	2.94	
Middle temporal gyrus	L	−60	−40	10	3.29	185
Superior temporal gyrus	L	−52	−40	12	3.04	
Postcentral gyrus	R	28	−42	58	3.22	167
Postcentral gyrus	R	36	−36	56	3.15	
Paracentral lobe	R	18	−44	50	2.80	
Superior temporal gyrus	R	58	−34	10	3.07	145
Middle temporal gyrus	R	56	−50	10	2.93	
Fusiform gyrus	R	28	−74	−6	3.03	135
Lingual gyrus	R	28	−50	−4	2.83	
Lingual gyrus	R	28	−58	−2	2.68	
*Commonalities in BOLD suppression for HC and SSD during real hand and bimodal trials*						
Middle occipital gyrus	L	−42	−72	16	3.91	5248
Calcarine	L	−4	−62	14	3.87	
Calcarine	L	−26	−68	10	3.79	
Superior temporal gyrus	L	−52	−40	12	3.42	252
Middle temporal gyrus	L	−48	−52	12	3.28	
Rolandic Operculum	L	−46	−30	22	3.03	
Fusiform gyrus	R	26	−80	−2	3.25	112
Supplementary motor area	L	−4	8	48	3.23	110
Supplementary motor area	L	−4	−2	52	2.78	
Middle frontal gyrus	L	−32	30	28	3.13	159
Middle frontal gyrus	L	−30	40	24	3.00	
Superior frontal gyrus	L	−28	48	22	2.78	
*Commonalities in BOLD suppression for HC and SSD during tool use and unimodal trials*						
Precuneus	R	6	−56	40	4.17	3553
Fusiform gyrus	L	−22	−80	−4	4.01	
Middle occipital gyrus	L	−22	−90	−2	3.82	
Middle cingulate cortex	L	−6	0	32	3.97	94
Superior temporal gyrus	R	58	−34	18	3.78	592
Supramarginal gyrus	R	64	−46	24	3.63	
Angular gyrus	R	62	−50	34	3.40	
Inferior frontal gyrus	L	−34	26	24	3.66	878
Inferior frontal gyrus	L	−50	22	18	3.65	
Inferior frontal gyrus	L	−36	40	6	3.58	
Precentral gyrus	L	−30	−8	58	3.66	285
Middle frontal gyrus	L	−30	4	50	3.29	
Superior frontal gyrus	L	−24	−2	48	3.28	
Superior temporal gyrus	L	−44	−34	20	3.56	125
Superior temporal gyrus	L	−54	−38	10	3.17	
Middle temporal gyrus	L	−42	−66	16	3.42	147
Middle occipital gyrus	L	−36	−72	22	3.00	
Superior frontal gyrus	R	22	46	30	3.35	117
Supplementary motor area	R	8	2	62	2.97	138
Superior frontal gyrus	R	18	12	56	2.87	
Superior frontal gyrus	R	22	4	58	2.85	
*Commonalities in BOLD suppression for HC and SSD during tool use and bimodal trials*						
Superior medial frontal gyrus	R	4	36	42	4.78	3812
Supplementary motor area	L	−6	18	56	4.38	
Supplementary motor area	L	0	10	54	4.03	
Inferior frontal gyrus	L	−36	28	22	4.52	3605
Inferior frontal gyrus	L	−50	22	22	4.10	
Middle frontal gyrus	L	−46	24	32	3.95	
Middle cingulate gyrus	L	−8	2	34	4.22	358
Middle cingulate cortex	R	4	0	30	3.53	
Middle cingulate cortex	R	4	−10	32	3.13	
Cerebellum, lobule 6	L	−10	−70	−22	4.18	493
Cerebellum, lobule VI	R	14	−68	−22	3.75	
Cerebellum, crus II	L	−16	−78	−34	3.11	
Fusiform gyrus	R	32	−64	−6	4.00	1825
Fusiform gyrus	L	−24	−74	−8	3.98	
Lingual gyrus	L	−18	−68	−2	3.70	
Superior parietal lobe	L	−18	−44	64	3.82	3303
Middle cingulate cortex	R	2	−42	42	3.78	
Angular gyrus	R	46	−68	42	3.64	
Superior temporal gyrus	L	−44	−36	20	3.82	257
Superior temporal gyrus	L	−54	−40	12	3.53	
Superior temporal gyrus	R	58	−36	20	3.73	632
Superior temporal gyrus	R	66	−34	22	3.64	
Superior temporal gyrus	R	52	−40	16	3.47	
Superior temporal gyrus	L	−44	−14	−4	3.71	228
Superior temporal gyrus	L	−48	−6	−6	3.67	
Superior temporal gyrus	L	−42	−6	−12	3.15	
Supplementary motor area	L	−6	−14	64	3.40	96

Whole brain results on group level are displayed. Coordinates are listed in MNI space. Significance threshold: *p* < 0.005 (unc.), with a cluster extent threshold of 94 voxels, *p* < 0.05 Monte Carlo cluster level corrected [37]. Source of anatomical labels: AAL3 toolbox for SPM12 [36]. *R* right, *L* left. N_SSD_ = 20, N_HC_ = 20.

Anatomical locations (peak voxels) were obtained from conjunction analyses identifying similar patterns of BOLD suppression (Passive > Active) for HC and SSD and for hand and tool use conditions, as well as for unimodal and bimodal trials, respectively.

#### Group differences

**Fig. 3:**
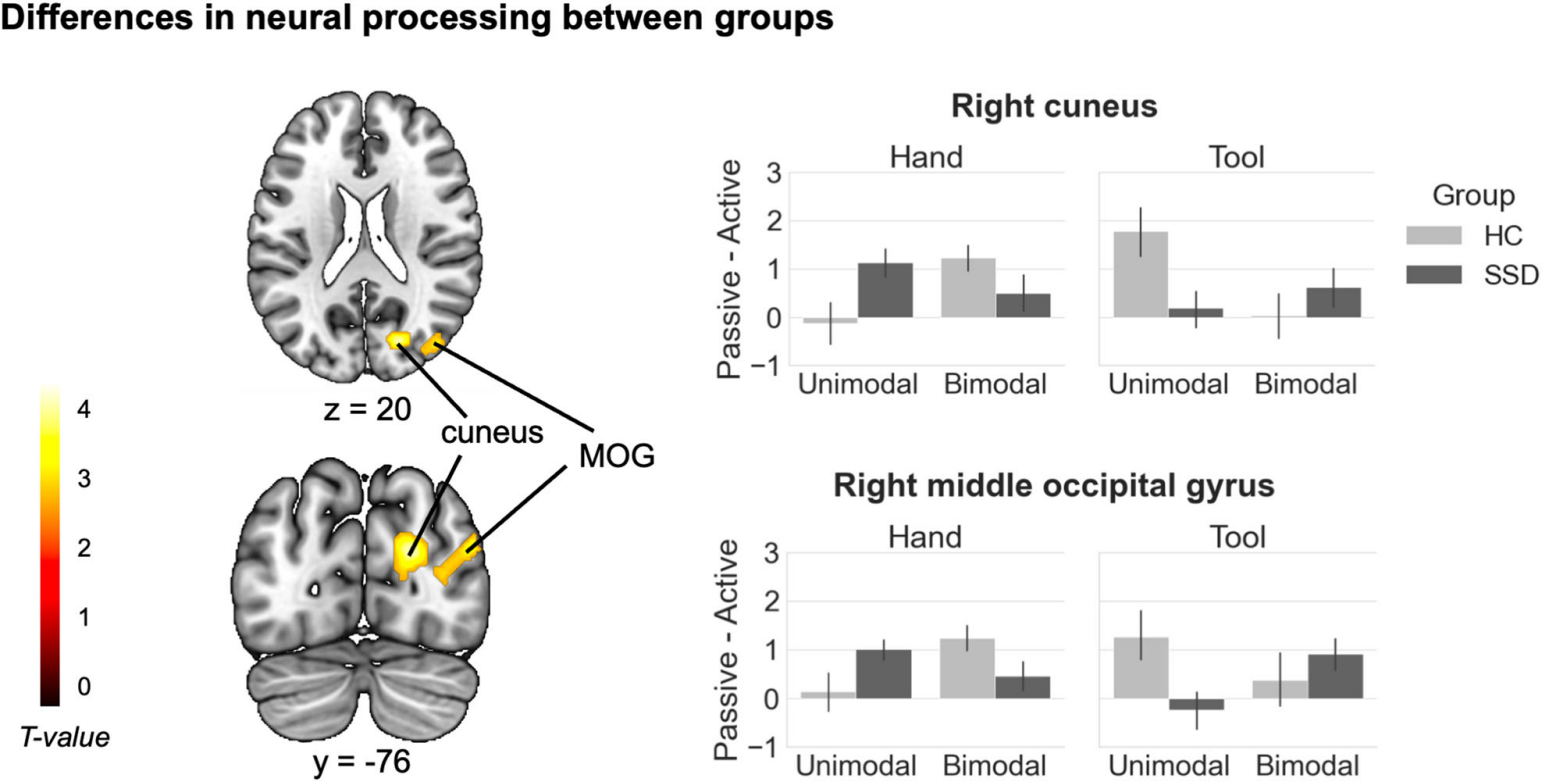
Differences between groups. Brain areas resulting from the four-way interaction analysis between the factors Group, Instrumentality, Movement type, and Modality are displayed. Significance threshold: *p* < 0.005 (unc.), with a cluster extent threshold of 94 voxels, *p* < 0.05 Monte Carlo cluster level corrected [37]. The bar graphs represent contrast estimates, which were extracted as eigenvariates from the two clusters with the respective peaks in the right cuneus and right middle occipital gyrus. Contrast estimates are displayed in terms of activity differences between actively and passively performed actions (Passive - Active) for each condition and group. Error bars show standard errors of the mean. *MOG* middle occipital gyrus, *R* right, *L* left. N_SSD_ = 20, N_HC_ = 20.

We found a significant interaction effect for Group (HC vs. SSD) x Instrumentality (hand vs. tool) x Movement type (active vs. passive) x Modality (unimodal vs. bimodal) in the right cuneus, extending to the right calcarine, and in the right middle occipital gyrus (see Fig. 3 and Table 3).

**Table 3:** Anatomical locations for the four-way interaction.

Anatomical Locations (Local Maxima)	Hemisphere	x	y	z	T	No. Voxels
Cuneus	R	20	−76	20	3.80	304
Cuneus	R	16	−82	26	3.15	
Calcarine	R	18	−84	12	2.72	
Middle occipital gyrus	R	46	−76	24	3.25	194
Middle occipital gyrus	R	32	−78	12	2.87	
Middle occipital gyrus	R	40	−74	38	2.78	

Whole brain results on group level are displayed. Coordinates are listed in MNI space. Significance threshold: *p* < 0.005 (unc.), with a cluster extent threshold of 94 voxels, *p* < 0.05 Monte Carlo cluster level corrected [37]. Source of anatomical labels: AAL3 toolbox for SPM12 [36]. *R* right, *L* left. N_SSD_ = 20, N_HC_ = 20.

Anatomical locations (peak voxels) were obtained from the interaction analysis between the factors Group, Instrumentality, Movement type, and Modality.

In HC, the activity difference between active and passive actions was stronger in bimodal than in unimodal trials in these regions during real hand actions, but stronger in unimodal trials during tool use actions. For SSD, the pattern was reversed, with a stronger active-passive difference in unimodal trials during real hand actions, and a stronger difference in bimodal trials during tool use actions. The inverse interaction contrast, as well as the two-way interactions between Group and Movement type did not reveal any suprathreshold activations.

## Discussion

We investigated similarities and differences between patients with SSD and HC regarding the processing of (delayed) unimodal or bimodal sensory input elicited by tool vs. hand movements. To manipulate the predictability of the sensory input, movements were either actively or passively performed. Both groups showed a similar pattern of BOLD suppression in visual processing regions (fusiform gyrus, middle occipital gyri), preparatory motor areas (SMA, cerebellum), and frontal regions (superior and middle frontal gyrus). Importantly, group differences emerged in right the cuneus, calcarine, and middle occipital gyrus, with reduced active-passive differences in patients, specifically for bimodal action feedback and tool use actions. Thus, although SSD and HC share commonalities when predicting the sensory consequences of actions, differential processes suggest dysfunctions in the underlying mechanisms in patients with SSD when tools or multimodal action feedback are involved.

### Group similarities

On a behavioral level, although patients with SSD had wider curves and thus showed a reduced ability to discriminate between the delay levels compared to HC, differences in detection performance between the experimental manipulations were evident across both groups. Both groups had smaller detection thresholds and thus detected more delays in passive than in active trials, indicating large similarities in behavioral suppression of self-generated action feedback – a result conflicting with our expectations and a series of earlier findings of impaired action feedback processing in SSD [5, 6].

At the neural level, both groups also showed large similarities, with BOLD suppression (i.e., less BOLD signal in active than passive conditions) for both tool and hand actions, as well as for unimodal and bimodal feedback. This was evident not only in visual processing regions (e.g., fusiform gyrus, middle occipital gyrus) but also in a variety of other regions, including frontal and temporoparietal ones (including precuneus), as well as the cerebellum and the SMA. Reduced activation of these regions during the processing of actively generated movement feedback, as opposed to passively elicited feedback, aligns well with previous findings on predictive processing in healthy subjects [2, 26, 39, 40]. Our findings thereby confirm that the processing of feedback elicited by real hand and tool use actions [25, 26, 41], as well as unimodal and bimodal feedback [2, 4], is based on similar predictive mechanisms. Thus, self-generated feedback in all these conditions was associated with largely similar patterns of BOLD suppression. These findings are also largely consistent with a previous fMRI study from our group using the same experimental design, in which processing hand and tool use actions in a sample of younger healthy subjects was likewise associated with widespread BOLD suppression in various frontal and temporoparietal regions, including the precuneus, fusiform gyrus, postcentral gyrus, and the cerebellum [26]. Importantly, we extend previous findings by showing that the suppression effect could be observed not only in HC but also in patients, even when it involved bimodal feedback or feedback generated by tool use actions. These commonalities suggest that predictive mechanisms based on the forward model are, to a certain extent, associated with similar neural processing in the mentioned regions in patients as in HC even for complex conditions where tools are used as an extension of the body.

### Group differences

Even though there were no differences in suppression between the groups at the behavioral level, at the neural level, we found a significant four-way interaction. For HC, the right cuneus, calcarine, and middle occipital gyrus yielded a differential pattern for active and passive as well as unimodal and bimodal conditions when subjects used their hand, and a reversed pattern when they used a tool. As part of the occipital lobe, the cuneus typically responds to visual stimuli and has been suggested to modulate signals from the primary visual cortex [42]. The middle occipital gyrus and the calcarine are also well-known areas for higher-level visual processing [43]. Furthermore, alongside the precuneus, the cuneus has been implicated in self-referential processing by contributing to the attribution of events or information to the self or to a different person [44–46]. This emphasizes that the differential representation of self-generated and externally generated sensory input emerges not only in primary sensory cortices but also in regions associated with higher-level sensory [7, 8, 18] and self-referential processing, especially in interaction with our experimental manipulations of action and feedback complexity. For real hand actions, differences in the neural representation of self- (actively) generated action feedback and externally (passively) generated sensory input in these regions appeared to be stronger in HC during bimodal compared to unimodal trials, similar to the behavioral results. This suggests that in healthy individuals, the advantage of action feedback processing of multiple sensory modalities [4, 47] modulates not only behavioral but also neural responses in the mentioned brain regions. For tool use actions, however, a different pattern emerged in HC, with a stronger active-passive difference in unimodal than in bimodal trials. It has already been demonstrated previously that the use of tools requires additional processing resources during action feedback perception [26]. This is because the tool shifts the location of the end effectors to a more distal point [48], and these altered sensorimotor mappings must be integrated into the action feedback prediction [26]. Thus, in our study, it is possible that the additional feedback complexity in bimodal trials, coupled with the use of the tool for performing the movement, led to a particularly high processing load, preventing the emergence of robust active-passive differences in the clusters of the interaction contrast. Together, the activation pattern observed in HC in the cuneus, calcarine, and middle occipital gyrus suggests that these regions differentiate between self- and externally generated sensory input and are thus involved in the processing of action feedback in situations where the feedback processing is characterized by an additional dimension of complexity, either in the action (e.g., through the use of a tool) or in the feedback itself (e.g., through bimodal feedback).

Compared to HC, patients with SSD did not show the facilitated active-passive differentiation in these visual regions during bimodal trials of real hand actions. Furthermore, during tool use actions, differences in BOLD signal for self- compared to externally generated sensory input were overall reduced in patients compared to HC. Aberrant processing in visual regions in SSD is already well documented. For instance, reduced activity in middle occipital gyrus and cuneus was observed in SSD compared to healthy individuals across various tasks [49]. It is also established that patients exhibit reduced gray matter volume in visual regions, including cuneus and calcarine [50], and reduced resting-state functional connectivity of calcarine [51], cuneus [52], and occipital cortex [53] with various cortical and subcortical regions. The gray matter volume [50] and connectivity strength [54] of these regions were even found to be negatively correlated with hallucination severity. This indicates severe anatomical and functional changes in these visual regions in SSD, which may represent an important substrate underlying the disorder and related symptomatology [55]. In this line, a previous study of our group demonstrated that neural suppression in the cuneus and middle occipital gyrus was reduced in patients compared to HC while they viewed video feedback of their hand being actively vs. passively moved [6]. This aligns well with the suggestion that disrupted processing in sensory regions contributes to the failure of predictive mechanisms based on the forward model in patients. For instance, reduced communication of these regions with various cortical and subcortical areas was suggested to result in the prediction about the sensory action consequences not being properly transmitted to sensory regions, preventing adequate modulation of their activity and consequently self-other distinction [56, 57]. In the present study, for HC, the right cuneus, calcarine, and middle occipital gyrus seemed to be primarily associated with the processing of action feedback characterized by additional complexity in the movement type or the feedback presentation. Thus, our results extend previous findings by demonstrating for the first time that these visual areas may fail to adequately use forward model predictions in patients to differentiate between self- and externally generated sensory input. As this primarily concerns complex action feedback scenarios in our study, especially those involving bimodal action feedback and feedback elicited by tool use actions, group differences might have occurred in these higher-level visual areas rather than in primary sensory areas.

### Limitations

There are some limitations that need to be acknowledged. The majority of patients presented only low to moderate symptomatology at the time of testing (see Table 1 in the main manuscript). This may partly explain the lack of differences in behavioral suppression effects between the groups since it has previously been shown that perceptual suppression is linked to the acute presence of hallucinations and/or ego disturbances rather than to an SSD diagnosis per se [58]. The low level of symptoms in the patient sample could also be partly responsible for the lack of correlations between symptom severity and neural suppression effects observed in our study (see S8 in the supplementary material). The results of the present study should therefore be interpreted with caution and should be validated in a larger sample size with more variability in symptom severity in the future. It is also conceivable that deficits in action feedback processing in patients are more likely to manifest in more complex tasks than the one applied in this study, such as in multimodal motor-cognitive tasks that require more processing resources, such as generating coherent thoughts and speech. Here, aberrant action feedback processing may have consequences at the neural and behavioral level which ultimately lead to psychopathological symptoms such as hallucinations and passivity phenomena. Lastly, it is also important to acknowledge the ongoing debate regarding whether predictive mechanisms based on a forward model are indeed responsible for the suppression effects observed in paradigms like the one used here, or whether more general sensorimotor gating mechanisms suppress all sensory input received during movement, regardless of whether it could be predicted or not [59]. Although the data from this study do not allow us to definitively distinguish between these two mechanisms, we believe there is evidence supporting the notion that the suppression effects are driven by an action-specific predictive mechanism, such as a forward model. For instance, similar neural suppression effects have been observed for abstract sensory outcomes that are perceived after the movement has been completed [39]. These effects cannot be explained by sensorimotor gating mechanisms, which are specifically active during movement execution. Furthermore, the probabilistic mapping between a movement and such an abstract outcome must be learned and even adapts when this mapping is repeatedly disrupted, for example, by a constant delay between the movement and the outcome, which shifts the suppression effect in time [60]. This phenomenon also requires an action-specific predictive mechanism. Ultimately, regardless of the exact mechanism at work in our paradigm, our results highlight both similarities and differences between patients and healthy controls during the processing of complex bimodal action feedback and tool use actions.

### Conclusions

Our findings show that neural activity elicited by unimodal and bimodal feedback of real hand and tool use actions is similarly suppressed in both patients with SSD and healthy individuals in a widespread network, including regions for visual processing, temporoparietal regions, as well as the cerebellum and SMA. However, particularly during the processing of bimodal action feedback and tool use actions, higher-level visual areas, including the cuneus, calcarine, and middle occipital gyrus, exhibited differences in differentiating between self- and externally generated sensory input in patients compared to HC. This demonstrates for the first time that dysfunctional predictive processes in these regions in SSD may underlie deficits in the processing of action feedback in complex everyday life situations with multimodal action feedback and when the action is mediated by the use of tools.

## Supplementary information

Supplementary material for the research article: Predictive neural processing of self-generated hand and tool actions in patients with schizophrenia spectrum disorders and healthy individuals

## Data Availability

The data that support the findings of this study are openly available in Zenodo at: 10.5281/zenodo.10943445.
