# Supplementary material for: Predictive neural processing of self-generated hand and tool actions in patients with schizophrenia spectrum disorders and healthy individuals
Source: Transl Psychiatry. 2025 Mar 17;15:85. doi: 10.1038/s41398-025-03306-6 (PMC11914148; doi:10.1038/s41398-025-03306-6)
Supplement: Supplementary file 1 — Supplementary material for the research article: Predictive neural processing of self-generated hand and tool actions in patients with schizophrenia spectrum disorders and healthy individuals [file 41398_2025_3306_MOESM1_ESM.docx]

**Supplementary material** for the research article: Predictive neural processing of self-generated hand and tool actions in patients with schizophrenia spectrum disorders and healthy individuals

## **S1. Exclusion criteria**

Exclusion criteria for both groups included a history of significant head trauma, neurological and general medical disorders potentially affecting brain physiology as well as current substance dependencies. Originally, 31 HC and 47 patients with SSD were included. Three HC and three SSD did not complete the study and one HC was excluded due to the report of hallucinatory experiences in the past. One patient was excluded due a prior history of epilepsy and another patient was excluded after the training session due to bad task performance and difficulties to understand the task. This resulted in the final sample of 27 HC and 42 patients which were matched for age, sex, and educational level (for details see **Table 1** in the main manuscript).

**S2. Group characteristics of the fMRI sample**

The group characteristics of the patients and HC that participated in the fMRI experiment are summarized in **Supplementary Table 1**.

**Supplementary Table 1. Group characteristics of the fMRI sample.**

|  | **SSD**  (fMRI sample;  N = 20) | **HC**  (fMRI sample;  N = 20) | **Group comparisons** | | |
| --- | --- | --- | --- | --- | --- |
|  |  |  | ***t*-value** | ***p*-value** | ***d*** |
| ***Demographics*** |  |  |  |  |  |
| Sex |  |  |  |  |  |
| Male | 17 (85%) | 15 (75%) |  |  |  |
| Female | 3 (15%) | 5 (25%) |  |  |  |
| Age (in years) | 38.00 ± 9.21  (21-53) | 38.40 ± 9.89  (20-55) | .132 | .895 | .042 |
| Education |  |  |  |  |  |
| Lower secondary | 14 (70%) | 13 (65%) |  |  |  |
| Upper secondary | 4 (20%) | 5 (25%) |  |  |  |
| Tertiary | 2 (10%) | 2 (10%) |  |  |  |
|  |  |  |  |  |  |
| ***Handedness*** |  |  |  |  |  |
| Laterality quotient (EHI) | 86.95 ± 17.45 (38.46-100) | 82.81 ± 29.28  (20-100) | -.543 | .590 | -.172 |
|  |  |  |  |  |  |
| ***Clinical measures*** |  |  |  |  |  |
| Positive symptoms  (SAPS total score) | **11.60 ± 10.30**  **(0-33)** | **1.85 ± 2.36**  **(0-9)** | **-4.129** | **< .001** | **-1.306** |
| Negative symptoms  (SANS total score) | **13.10 ± 11.93**  **(0-35)** | **1.30 ± 2.00**  **(0-8)** | **-4.363** | **< .001** | **-1.380** |
| ***Neuropsychological control measures*** | | | | | |
| **Attention (d2)** |  |  |  |  |  |
| Number of items worked on | 421.58 ± 104.45 (211-586)^a^ | 461.20 ± 94.30 (317-639) | 1.245 | .221 | .399 |
| Total error score | 18.00 ± 14.62  (1-61)^a^ | 20.25 ± 13.81  (3-55) | .494 | .624 | .158 |
| **Executive functions** |  |  |  |  |  |
| TMT A (in s) | 29.30 ± 7.26  (18-41) | 30.95 ± 17.11  (16-84) | .397 | .694 | .126 |
| TMT B (in s) | 78.35 ± 30.39  (31-139) | 64.40 ± 29.15  (27-157) | -1.481 | .147 | -.468 |
| **Intelligence (MWT-B-IQ)** | 103.60 ± 13.67  (79-143) | 103.90 ± 14.19  (85-136) | .068 | .946 | .126 |
| **Short term memory (WAIS-DS)** |  |  |  |  |  |
| Forward | 9.65 ± 1.39  (7-13) | 9.25 ± 1.65  (7-13) | -.830 | .412 | -.262 |
| Backward | 8.00 ± 1.52  (5-10) | 8.33 ± 1.64  (5-12) | .395 | .695 | .125 |
|  |  |  |  |  |  |
| ***Antipsychotic medication*** |  |  |  |  |  |
| None | 3 | 20 |  |  |  |
| First generation | 3^b^ | 0 |  |  |  |
| Second generation | 16^b^ | 0 |  |  |  |

*Note*: ^a^ N = 19, ^b^ Two patients were treated with both first and second generation antipsychotics.
d2 = d2 Test of Attention,^1^ EHI = Edinburgh Handedness Inventory,^2^ MWT-B = Multiple choice vocabulary test,^3^ SANS = Scale for the Assessment of Negative Symptoms,^4^ SAPS = Sale for the Assessment of Positive Symptoms,^5^ TMT = Trail Making Test,^6^ WAIS-DS = Wechsler Adult Intelligence Scale - Digit Span.^7^ Data for continuous variables are reported as mean ± standard deviation (range). For categorical data, the percentage of cases in each category is shown in brackets. Potential differences between groups in age, handedness, clinical scores, and neuropsychological control measures were assessed by independent-samples t-tests. The corresponding t-values, p-values and Cohen’s d are provided for each test. Significant group differences are presented with bold values (*p* < .05, uncorrected).

## **S3. Movement device**

The custom-made MR-compatible movement device is depicted in **Supplementary Fig. 1**. The movement device could be moved along a circular arc from a starting position on the left to a turning point on the right and back. Subjects moved the handle of the device either with their own hand or they used a custom-made tool which was mounted onto the handle. They held onto the tool with a whole hand grip and performed the same hand movement to move it.


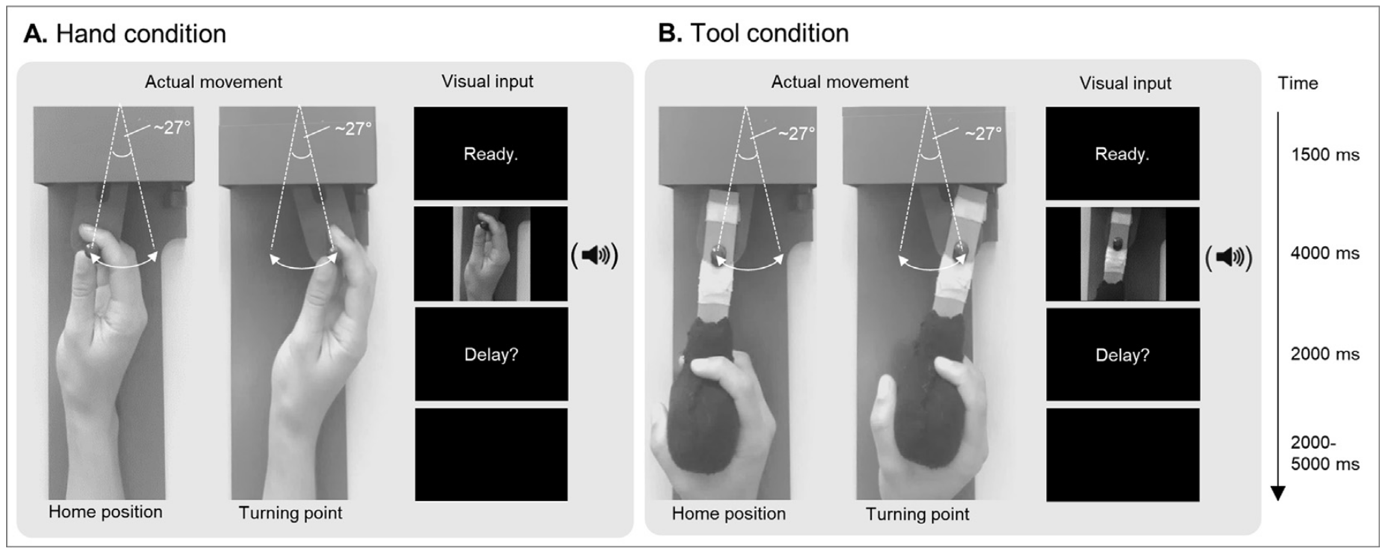


**Supplementary Fig. 1. Depiction of the experimental setup in hand and tool use conditions.** Subjects grabbed and moved the handle of the device with their own hand **(A)** or they moved the handle via a tool **(B)**. In conditions with unimodal feedback, they received visual action feedback in form of a real-time video feedback of their hand movement. In bimodal feedback conditions, the video was accompanied by a sound which was played when the handle reached the turning point. Data from healthy subjects using the same experimental setup have been published earlier.^8^

## **S4. Details on the experimental procedure**

Before the beginning of each of the four runs, subjects were informed about whether movements had to be performed with their hand or with the tool and the experimenter added or removed the tool accordingly. Movements were performed with the same instrument (hand or tool) for two consecutive runs. Whether the experiment started with the hand or tool condition was counterbalanced across subjects. In each run, active and passive conditions were blocked and announced by written instructions on the screen at the beginning of each block. Whether a run started with an active or passive block was assigned randomly. Within each block, an equal number of bimodal and unimodal trials were presented in pseudorandom order.

Each trial started with the cue “Ready” presented for 1500ms on the screen. Afterwards the camera image was displayed for a duration of 4000ms. In active conditions, movements could be performed with the right hand within that time window whenever subjects felt ready to move. In passive conditions, movements were performed by the device 500ms after onset of the camera image which remained visible for the entire period of 4000ms. After the camera image disappeared, subjects had to respond via key press with their left hand on a MR-compatible button pad (Cedrus, Lumina, San Pedro, USA) whether they detected a delay between the movement and the (audio-)visual feedback (yes or no). The button assignment was counterbalanced across subjects. Subjects were instructed to respond as accurately but not as fast as possible. Each trial ended with an inter-trial interval (blank screen for 2000-5000ms).

Subjects were also invited to a training session outside the MRI scanner prior to the fMRI experiment to familiarize themselves with the task. During the training session, subjects were seated in front of a monitor and put their right forearm onto a table to operate the movement device. A curtain obscured the view of the forearm and hand. The experimenter’s verbal instructions focused on the correct execution of the active movement until subjects used the correct grip on the device’s handle and moved it entirely to the turning point and back. A metronome was used to train subjects to execute the movements at the correct speed (total duration of ~1.5s per movement). For passive movements, subjects were told to relax their wrist while holding on to the handle. They were then familiarized with the trial sequence during the delay detection task, which initially provided on-screen information about whether a delay had been present (yes or no) at the end of each trial. Lastly, they completed one run in which the trial sequence was equal to the main experimental runs. Instructions were identical for the hand and the tool condition, allowing both conditions to be rehearsed to an equal extent. The same experimental training procedure has been applied before with healthy subjects.^8^

## **S5. Sanity check**

Prior to the analysis of behavioral data, we conducted a sanity check to determine whether subjects were able to perform the delay detection task adequately. To this end, we computed the average length of delay for trials in which subjects did vs. did not detect a delay (i.e., for “yes” vs. “no” responses, respectively). Since it is impossible to consistently detect smaller delays while not detecting larger delays, the average delay should be greater for “yes” compared to “no” trials. For four subjects (1 HC and 3 SSD), this was not the case in any of the experimental conditions, indicating that they mixed up the button assignment. Therefore, we flipped the responses for these four participants (i.e., “yes” became “no” and vice versa).

**S6. Detailed summaries of behavioral statistical results**

To evaluate delay detection performances, psychometric functions were fitted to the delay detection data and detection thresholds and widths of the functions were extracted as summary measures. Results of mixed ANOVAs on these measures and the corresponding inclusion Bayes factors for each effect are summarized in **Supplementary Tables 2 and 3**. Significant effects are indicated with bold values.

**Supplementary Table 2. Results of the ANOVA on detection thresholds.**

| Effect | df | F | p | η_p_^2^ | BF_incl_ |
| --- | --- | --- | --- | --- | --- |
| Group | 1 | 2.023 | 0.160 | 0.029 | 0.152 |
| *Residuals* | 67 |  |  |  |  |
| Instrumentality | 1 | 2.009 | 0.161 | 0.029 | 0.115 |
| Instrumentality ✻ Group | 1 | 0.063 | 0.802 | 9.435e -4 | 0.079 |
| *Residuals* | 67 |  |  |  |  |
| **Movement type** | **1** | **15.994** | **< .001** | **0.193** | **1227.016** |
| Movement type ✻ Group | 1 | 0.524 | 0.472 | 0.008 | 0.140 |
| *Residuals* | 67 |  |  |  |  |
| Modality | 1 | 3.018 | 0.087 | 0.043 | 0.058 |
| Modality ✻ Group | 1 | 1.627 | 0.207 | 0.024 | 0.080 |
| *Residuals* | 67 |  |  |  |  |
| Instrumentality ✻ Movement type | 1 | 0.661 | 0.419 | 0.010 | 0.082 |
| Instrumentality ✻ Movement type ✻ Group | 1 | 0.382 | 0.539 | 0.006 | 0.001 |
| *Residuals* | 67 |  |  |  |  |
| Instrumentality ✻ Modality | 1 | 0.236 | 0.629 | 0.004 | 0.014 |
| Instrumentality ✻ Modality ✻ Group | 1 | 0.266 | 0.608 | 0.004 | 2.943e-4 |
| *Residuals* | 67 |  |  |  |  |
| Movement type ✻ Modality | 1 | 3.406 | 0.069 | 0.048 | 0.099 |
| Movement type ✻ Modality ✻ Group | 1 | 0.823 | 0.368 | 0.012 | 0.003 |
| *Residuals* | 67 |  |  |  |  |
| Instrumentality ✻ Movement type ✻ Modality | 1 | 0.095 | 0.759 | 0.001 | 0.001 |
| Instrument ✻ Movement type ✻ Modality ✻ Group | 1 | 0.468 | 0.496 | 0.007 | 0.007 |
| *Residuals* | 67 |  |  |  |  |

N_SSD_ = 42, N_HC_ = 27

**Supplementary Table 3. Results of the ANOVA on the widths of the psychometric functions.**

| Effect | df | F | p | η_p_^2^ | BF_incl_ |
| --- | --- | --- | --- | --- | --- |
| **Group** | **1** | **11.234** | **0.001** | **0.144** | **3.860** |
| *Residuals* | 67 |  |  |  |  |
| Instrumentality | 1 | 2.804 | 0.099 | 0.040 | 0.064 |
| Instrumentality ✻ Group | 1 | 1.597 | 0.211 | 0.023 | 0.081 |
| *Residuals* | 67 |  |  |  |  |
| **Movement type** | **1** | **13.781** | **< .001** | **0.171** | **765.365** |
| Movement type ✻ Group | 1 | 0.380 | 0.540 | 0.006 | 0.166 |
| *Residuals* | 67 |  |  |  |  |
| Modality | 1 | 2.825 | 0.097 | 0.040 | 0.096 |
| Modality ✻ Group | 1 | 0.013 | 0.910 | 1.904e -4 | 0.052 |
| *Residuals* | 67 |  |  |  |  |
| Instrumentality ✻ Movement type | 1 | 0.246 | 0.622 | 0.004 | 0.052 |
| Instrumentality ✻ Movement type ✻ Group | 1 | 0.038 | 0.846 | 5.681e -4 | 0.002 |
| *Residuals* | 67 |  |  |  |  |
| Instrumentality ✻ Modality | 1 | 0.176 | 0.676 | 0.003 | 0.018 |
| Instrumentality ✻ Modality ✻ Group | 1 | 1.100 | 0.298 | 0.016 | 0.001 |
| *Residuals* | 67 |  |  |  |  |
| Movement type ✻ Modality | 1 | 1.426 | 0.237 | 0.021 | 0.074 |
| Movement type ✻ Modality ✻ Group | 1 | 0.135 | 0.715 | 0.002 | 0.002 |
| *Residuals* | 67 |  |  |  |  |
| Instrumentality ✻ Movement type ✻ Modality | 1 | 0.010 | 0.920 | 1.503e -4 | 4.955e-4 |
| Instrumentality ✻ Movement type ✻ Modality ✻ Group | 1 | 0.212 | 0.647 | 0.003 | 1.244e-7 |
| *Residuals* | 67 |  |  |  |  |

 N_SSD_ = 42, N_HC_ = 27

**S7. Control analyses excluding trials with errors in movement execution**

Due to occasional problems with the algorithm of the movement device or because subjects did not execute the movements correctly, in some bimodal trials the sound may not have been audible. To make sure that the results reported in the main manuscript were not affected by this technical issue, the analysis of fMRI and behavioral data were repeated while excluding all bimodal trials for which the movement was logged as erroneous.

***Behavioral data***

For the analysis of behavioral data excluding bimodal trials with movement errors, the data of one patient, and one run for another patient had to be excluded. For the remaining subjects and runs 2.33% of all trials had to be excluded for HC, and 4.34% of all trials for SSD. The results of this analysis are summarized in **Supplementary Fig. 2**. The mixed ANOVA on the detection thresholds yielded largely similar results as the same ANOVA including movement errors reported in the main manuscript, with a significant main effect of Movement type [*F*(1, 66) = 15.791, *p* < .001, η_p_^2^ = .193, BF_incl_ = 1108.658; Active: *M* = 271.110, *SE* = 13.210; Passive: *M* = 248.111, *SE* = 13.452]. As in the analysis reported in the main manuscript, none of the other main or interaction effects reached significance (all *p* > .056, all BF_incl_ < .159).

The mixed ANOVA on the widths of the psychometric functions revealed similar results for all effects as the analysis reported in the main manuscript, with significant main effects of Group [*F*(1, 66) = 11.992, *p* < .001, η_p_^2^ = .154, BF_incl_ = 5.309; SSD: *M* = 332.891, *SE* = 18.580; HC: *M* = 245.156, *SE* = 13.263] and Movement type [*F*(1, 66) = 12.192, *p* < .001, η_p_^2^ = .156, BF_incl_ = 557.882; Active: *M* = 270.092, *SE* = 13.095; Passive: *M* = 326.019, *SE* = 17.259]. None of the other main or interaction effects were significant (all *p* > .060, all BF_incl_ < .216). See **Supplementary Tables 4 and 5** for summaries of all effects.

***fMRI data***

For the analysis of fMRI data excluding bimodal trials with movement errors, the data of one patient, and one run for another patient had to be excluded. For the remaining subjects and runs 2.34% of all trials had to be excluded for HC, and 3.45% of all trials for SSD. The contrasts reported in the main manuscript were associated with very similar activation pattern for both analyses, whether excluding or including trials with movement errors. This was the case for the conjunctions of the Passive > Active contrast for both groups calculated for each of the four remaining experimental conditions (HandUni, HandBi, ToolUni, ToolBi; see **Supplementary Fig. 3** and **Supplementary Table 6**), as well as for the four-way interaction including the factors Group, Instrumentality, Movement type, and Modality (see **Supplementary Fig. 4** and **Supplementary Table 6**).

To conclude, due to the similarity of the fMRI and behavioral analyses excluding and including movement errors, it can be argued that the results reported in the main manuscript were not corrupted by the mentioned technical issues. Excluding the erroneous trials would not lead to different conclusions.


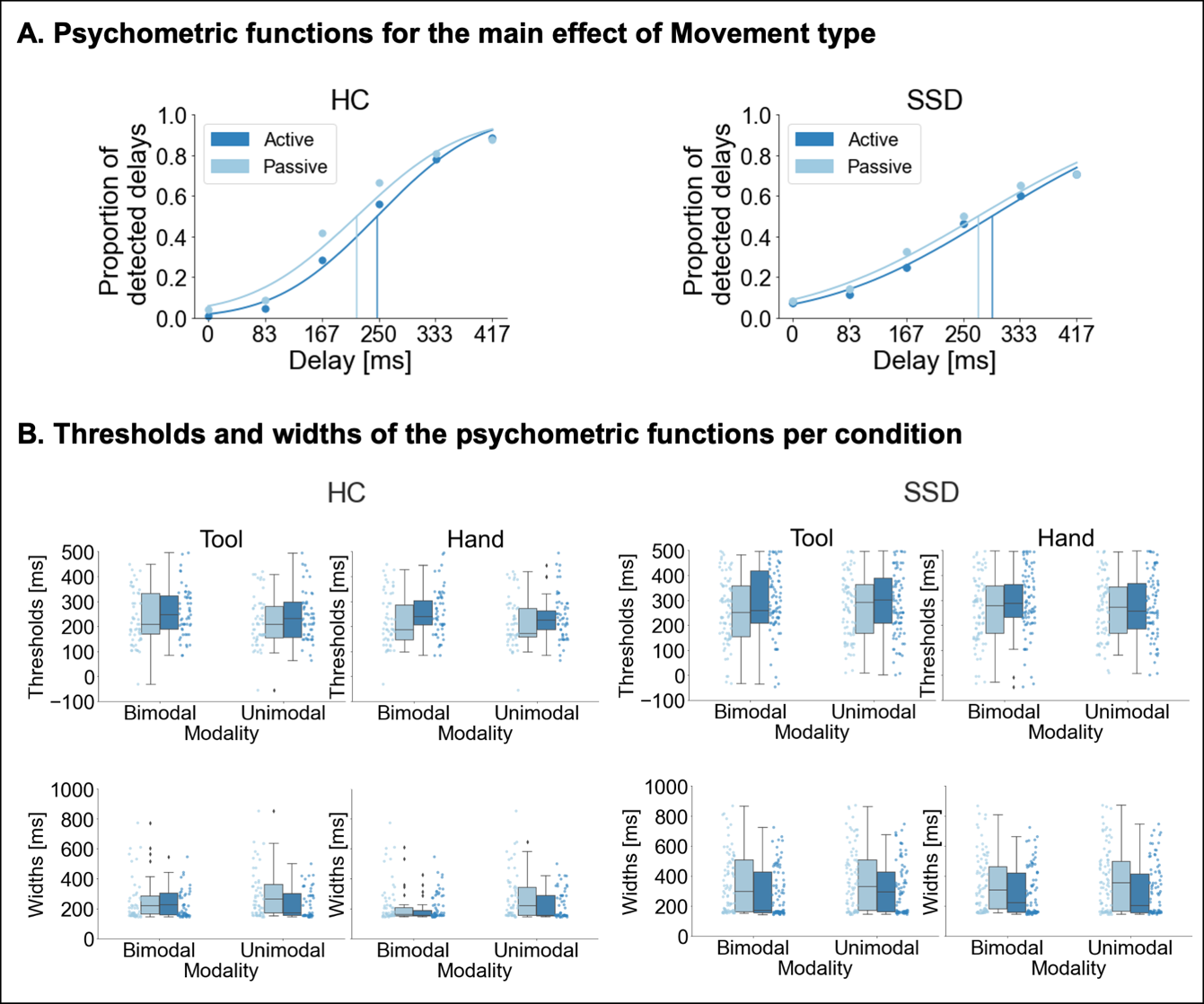


Supplementary Fig. 2. Behavioral results obtained by the analysis excluding movement errors in bimodal trials. A: Psychometric functions are displayed here for active and passive conditions and separately for both groups. B. Thresholds and widths of the psychometric functions are displayed for each individual condition. N_SSD_ = 41, N_HC_ = 27.

**Supplementary Table 4. Results of the ANOVA on detection thresholds excluding movement errors in bimodal trials.**

| Effect | df | F | p | η_p_^2^ | BF_incl_ |
| --- | --- | --- | --- | --- | --- |
| Group | 1 | 2.464 | 0.121 | 0.036 | 0.158 |
| *Residuals* | 66 |  |  |  |  |
| Instrumentality | 1 | 1.523 | 0.221 | 0.023 | 0.061 |
| Instrumentality ✻ Group | 1 | 0.133 | 0.717 | 0.002 | 0.027 |
| *Residuals* | 66 |  |  |  |  |
| **Movement type** | **1** | **15.791** | **< .001** | **0.193** | **1108.658** |
| Movement type ✻ Group | 1 | 0.360 | 0.550 | 0.005 | 0.090 |
| *Residuals* | 66 |  |  |  |  |
| Modality | 1 | 3.739 | 0.057 | 0.054 | 0.070 |
| Modality ✻ Group | 1 | 1.403 | 0.240 | 0.021 | 0.040 |
| *Residuals* | 66 |  |  |  |  |
| Instrumentality ✻ Movement type | 1 | 0.867 | 0.355 | 0.013 | 0.070 |
| Instrumentality ✻ Movement type ✻ Group | 1 | 0.349 | 0.557 | 0.005 | 0.001 |
| *Residuals* | 66 |  |  |  |  |
| Instrumentality ✻ Modality | 1 | 0.027 | 0.870 | 4.100e-4 | 0.014 |
| Instrumentality ✻ Modality ✻ Group | 1 | 0.484 | 0.489 | 0.007 | 3.074e-4 |
| *Residuals* | 66 |  |  |  |  |
| Movement type ✻ Modality | 1 | 3.488 | 0.066 | 0.050 | 0.146 |
| Movement type ✻ Modality ✻ Group | 1 | 1.062 | 0.306 | 0.016 | 0.004 |
| *Residuals* | 66 |  |  |  |  |
| Instrumentality ✻ Movement type ✻ Modality | 1 | 0.010 | 0.920 | 1.529e-4 | 0.001 |
| Instrumentality ✻ Movement type ✻ Modality ✻ Group | 1 | 0.717 | 0.400 | 0.011 | 2.232e-7 |
| *Residuals* | 66 |  |  |  |  |

 N_SSD_ = 41, N_HC_ = 27

**Supplementary Table 5. Results of the ANOVA on the widths of the psychometric functions excluding movement errors in bimodal trials.**

| Effect | df | F | p | η_p_^2^ | BF_incl_ |
| --- | --- | --- | --- | --- | --- |
| **Group** | **1** | **11.992** | **< .001** | **0.154** | **5.309** |
| *Residuals* | 66 |  |  |  |  |
| Instrumentality | 1 | 3.327 | 0.073 | 0.048 | 0.078 |
| Instrumentality ✻ Group | 1 | 1.499 | 0.225 | 0.022 | 0.089 |
| *Residuals* | 66 |  |  |  |  |
| **Movement type** | **1** | **12.192** | **< .001** | **0.156** | **557.882** |
| Movement type ✻ Group | 1 | 0.612 | 0.437 | 0.009 | 0.215 |
| *Residuals* | 66 |  |  |  |  |
| Modality | 1 | 3.641 | 0.061 | 0.052 | 0.192 |
| Modality ✻ Group | 1 | 0.002 | 0.967 | 2.651e-5 | 0.080 |
| *Residuals* | 66 |  |  |  |  |
| Instrumentality ✻ Movement type | 1 | 0.083 | 0.774 | 0.001 | 0.052 |
| Instrumentality ✻ Movement type ✻ Group | 1 | 0.003 | 0.958 | 4.281e-5 | 0.003 |
| *Residuals* | 66 |  |  |  |  |
| Instrumentality ✻ Modality | 1 | 0.322 | 0.572 | 0.005 | 0.033 |
| Instrumentality ✻ Modality ✻ Group | 1 | 1.025 | 0.315 | 0.015 | 0.002 |
| *Residuals* | 66 |  |  |  |  |
| Movement type ✻ Modality | 1 | 1.875 | 0.176 | 0.028 | 0.129 |
| Movement type ✻ Modality ✻ Group | 1 | 0.370 | 0.545 | 0.006 | 0.020 |
| *Residuals* | 66 |  |  |  |  |
| Instrumentality ✻ Movement type ✻ Modality | 1 | 0.228 | 0.634 | 0.003 | 8.847e-4 |
| Instrumentality ✻ Movement type ✻ Modality ✻ Group | 1 | 0.023 | 0.881 | 3.429e-4 | 2.106e-7 |
| *Residuals* | 66 |  |  |  |  |

 N_SSD_ = 41, N_HC_ = 27


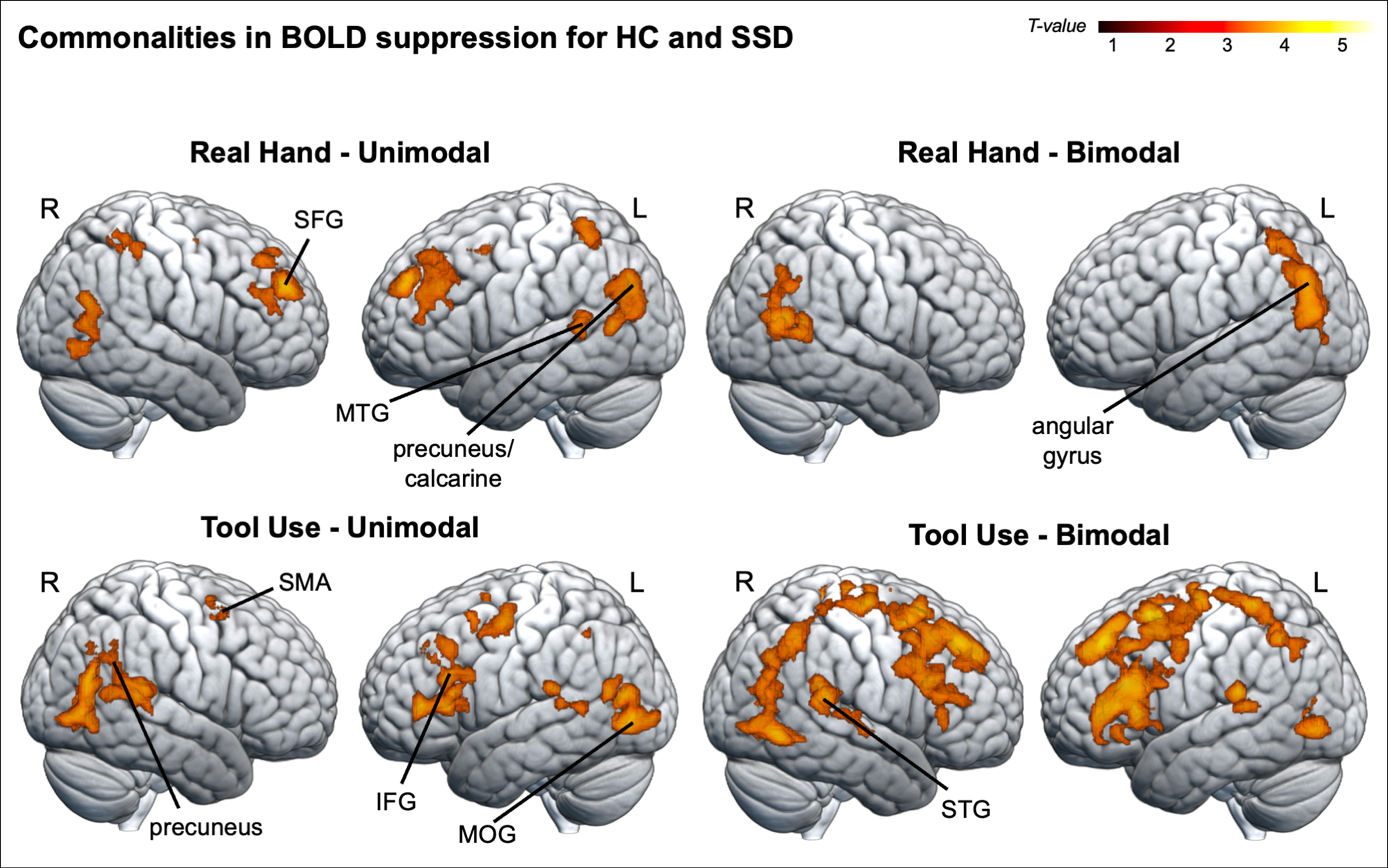


**Supplementary Fig. 3. Commonalities in BOLD suppression for SSD and HC obtained by the analysis excluding movement errors in bimodal trials.** Commonalities in BOLD suppression (less BOLD signal in active compared to passive conditions) for HC and SSD were assessed by means of conjunction analyses for each of the remaining four experimental conditions (HandUni, HandBi, ToolUni, ToolBi). Significance threshold: *p* < .005 (unc.) with a cluster extent threshold of 94 voxels.^9^ MFG = middle frontal gyrus, MTG = middle temporal gyrus, SFG = superior frontal gyrus, R = right, L = left. N_SSD_ = 19, N_HC_ = 20.


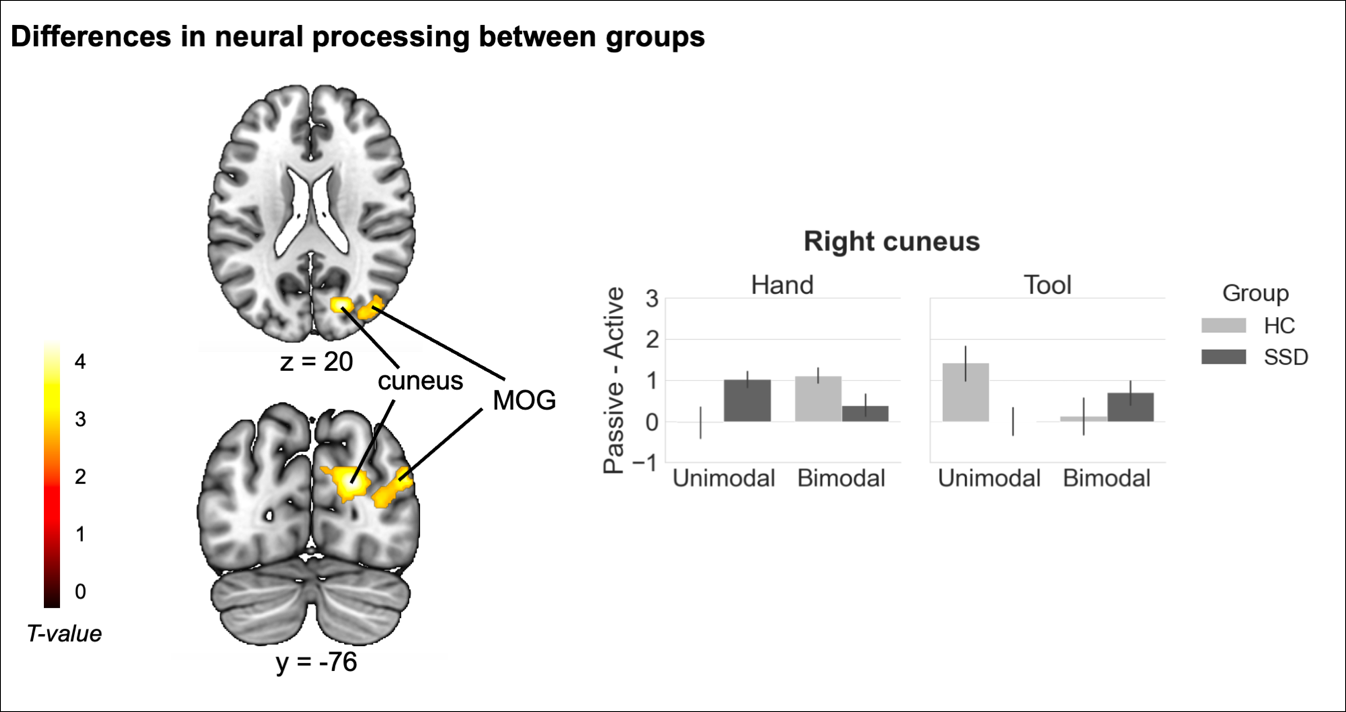


Supplementary Fig. 4. Differences between groups

**obtained by the analysis excluding movement errors in bimodal trials.** Brain areas resulting from the four-way interaction analysis between the factors Group, Instrumentality, Movement type, and Modality are displayed. Significance threshold: *p* < .005 (unc.) with a cluster extent threshold of 94 voxels, *p* < .05 Monte Carlo cluster level corrected.^9^ The bar graphs represent contrast estimates that were extracted as eigenvariates from the cluster with peak in the right cuneus. Contrast estimates are displayed in terms of activity differences between actively and passively performed actions (Passive - Active) for each condition and group. Error bars show standard errors of the mean. MOG = middle occipital gyrus, R = right, L = left. N_SSD_ = 19, N_HC_ = 20.

**Supplementary Table 6. Anatomical locations for the conjunction and interaction analyses.**

Anatomical locations (peak voxels) obtained from the conjunction and interaction analyses reported in the main manuscript but excluding all trials with movement errors.

| Anatomical Locations (Local Maxima) | Hemisphere | x | y | z | T | No. Voxels |
| --- | --- | --- | --- | --- | --- | --- |
| *Commonalities in BOLD suppression for HC and SSD during real hand and unimodal trials* | | | | | | |
| Superior medial frontal gyrus | R | 4 | 50 | 28 | 3.84 | 460 |
| Superior medial frontal gyrus | R | 4 | 36 | 44 | 3.01 |  |
| Precuneus | L | -8 | -60 | 14 | 3.73 | 1623 |
| Precuneus | R | 6 | -60 | 26 | 3.70 |  |
| Precuneus | L | -2 | -62 | 26 | 3.43 |  |
| Middle occipital gyrus | R | 42 | -64 | 24 | 3.46 | 279 |
| Middle temporal gyrus | R | 44 | -66 | 12 | 3.18 |  |
| Middle temporal gyrus | R | 54 | -68 | 14 | 2.91 |  |
| Middle occipital gyrus | L | -42 | -72 | 32 | 3.45 | 635 |
| Middle temporal gyrus | L | -44 | -62 | 8 | 3.41 |  |
| Middle occipital gyrus | L | -46 | -78 | 18 | 3.13 |  |
| Middle frontal gyrus | L | -30 | 34 | 28 | 3.28 | 507 |
| Middle frontal gyrus | L | -28 | 42 | 28 | 3.10 |  |
| Superior frontal gyrus | L | -18 | 34 | 44 | 3.04 |  |
| Superior temporal gyrus | L | -64 | -42 | 12 | 3.26 | 152 |
| Superior temporal gyrus | L | -52 | -40 | 12 | 2.80 |  |
| Supplementary motor area | L | -6 | 4 | 48 | 3.24 | 139 |
| Supplementary motor area | R | 2 | -6 | 52 | 2.94 |  |
| Supplementary motor area | L | -6 | 16 | 48 | 2.63 |  |
| Fusiform gyrus | R | 26 | -74 | -4 | 3.14 | 100 |
| Fusiform gyrus | R | 28 | -66 | -4 | 2.98 |  |
| Middle frontal gyrus | R | 28 | 40 | 28 | 3.00 | 105 |
| Superior frontal gyrus | R | 28 | 46 | 18 | 2.84 |  |
| Postcentral gyrus | R | 36 | -36 | 56 | 2.99 | 147 |
| Postcentral gyrus | R | 28 | -42 | 58 | 2.98 |  |
| Postcentral gyrus | R | 24 | -48 | 62 | 2.83 |  |
| *Commonalities in BOLD suppression for HC and SSD during real hand and bimodal trials* | | | | | | |
| Calcarine | L | -4 | -62 | 14 | 3.77 | 4067 |
| Angular gyrus | L | -38 | -68 | 36 | 3.60 |  |
| Calcarine | L | -26 | -68 | 10 | 3.59 |  |
| *Commonalities in BOLD suppression for HC and SSD during tool use and unimodal trials* | | | | | | |
| Precuneus | R | 6 | -62 | 26 | 3.84 | 2085 |
| Precuneus | R | 6 | -56 | 40 | 3.81 |  |
| Middle occipital gyrus | R | 34 | -68 | 16 | 3.81 |  |
| Fusiform gyrus | L | -22 | -80 | -4 | 3.80 | 850 |
| Calcarine | L | -20 | -76 | 10 | 3.72 |  |
| Inferior occipital gyrus | L | -24 | -88 | -4 | 3.34 |  |
| Insula | L | -32 | 26 | 8 | 3.74 | 905 |
| Inferior frontal gyrus | L | -36 | 40 | 8 | 3.69 |  |
| Inferior frontal gyrus | L | -36 | 26 | 22 | 3.57 |  |
| Superior temporal gyrus | R | 56 | -36 | 18 | 3.70 | 511 |
| Middle temporal gyrus | R | 52 | -52 | 16 | 3.27 |  |
| Inferior parietal lobe | R | 58 | -56 | 38 | 3.17 |  |
| Superior temporal gyrus | L | -44 | -34 | 18 | 3.43 | 146 |
| Superior temporal gyrus | L | -56 | -38 | 10 | 3.11 |  |
| Middle temporal gyrus | L | -50 | -50 | 8 | 3.02 |  |
| Middle temporal gyrus | L | -42 | -66 | 16 | 3.43 | 125 |
| Middle occipital gyrus | L | -36 | -72 | 22 | 2.97 |  |
| Precentral gyrus | L | -30 | -8 | 56 | 3.42 | 213 |
| Middle frontal gyrus | L | -30 | 4 | 50 | 3.04 |  |
| Superior frontal gyrus | L | -24 | -2 | 48 | 3.01 |  |
| Supplementary motor area | L | -6 | 10 | 54 | 3.11 | 135 |
| Supplementary motor area | R | 12 | 0 | 64 | 2.90 |  |
| Supplementary motor area | R | 8 | 10 | 56 | 2.83 |  |
| *Commonalities in BOLD suppression for HC and SSD during tool use and bimodal trials* | | | | | | |
| Inferior frontal gyrus | L | -36 | 28 | 22 | 4.31 | 3752 |
| Inferior frontal gyrus | L | -48 | 22 | 24 | 3.85 |  |
| Insula | L | -34 | 24 | 10 | 3.83 |  |
| Supplementary motor area | L | -6 | 14 | 56 | 4.27 | 2923 |
| Superior medial frontal gyrus | R | 2 | 40 | 40 | 3.97 |  |
| Supplementary motor area | R | 12 | -14 | 66 | 3.79 |  |
| Middle cingulate cortex | L | -6 | 2 | 32 | 4.09 | 363 |
| Middle cingulate cortex | R | 4 | -2 | 30 | 3.65 |  |
| Middle cingulate cortex | R | 6 | -10 | 34 | 3.13 |  |
| Superior temporal gyrus | L | -44 | -36 | 20 | 3.87 | 208 |
| Superior temporal gyrus | L | -54 | -38 | 12 | 3.43 |  |
| Fusiform gyrus | R | 34 | -62 | -8 | 3.77 | 1397 |
| Angular gyrus | R | 46 | -70 | 40 | 3.53 |  |
| Lingual gyrus | R | 20 | -70 | 0 | 3.36 |  |
| Superior temporal gyrus | R | 58 | -36 | 20 | 3.61 | 321 |
| Supramarginal gyrus | R | 66 | -34 | 24 | 3.57 |  |
| Supramarginal gyrus | R | 66 | -40 | 32 | 3.41 |  |
| Inferior occipital gyrus | L | -26 | -80 | -6 | 3.48 | 464 |
| Lingual gyrus | L | -14 | -70 | 0 | 3.45 |  |
| Middle occipital gyrus | L | -36 | -74 | 2 | 2.81 |  |
| Precentral gyrus | L | -44 | -2 | 44 | 3.40 | 95 |
| Precentral gyrus | L | -42 | 0 | 36 | 2.77 |  |
| Inferior parietal lobe | R | 30 | -52 | 50 | 3.35 | 350 |
| Angular gyrus | R | 28 | -58 | 40 | 3.17 |  |
| Postcentral gyrus | R | 22 | -36 | 64 | 2.84 |  |
| Angular gyrus | L | -42 | -70 | 46 | 3.16 | 104 |
| Angular gyrus | L | -40 | -66 | 38 | 2.93 |  |
| Heschl’s gyrus | R | 46 | -22 | 6 | 3.06 | 102 |
| Superior temporal gyrus | R | 52 | -14 | 0 | 3.04 |  |
| *Interaction of the factors Group, Movement type, Instrumentality, and Modality* | | | | | | |
| Cuneus | R | 20 | -76 | 22 | 4.05 | 680 |
| Middle occipital gyrus | R | 46 | -78 | 22 | 3.73 |  |
| Middle occipital gyrus | R | 32 | -78 | 14 | 3.02 |  |

*Note.* Whole brain results on group level are displayed. Coordinates are listed in MNI space. Significance threshold: *p* < .005 (unc.) with a cluster extent threshold of 94 voxels, *p* < .05 Monte Carlo cluster level corrected.^9^ Source of anatomical labels: AAL3 toolbox for SPM12.^10^ R = right, L = left. N_SSD_ = 19,
N_HC_ = 20.

**S8. Exploratory correlation analyses between chlorpromazine (CPZ) equivalents and the main behavioral and neural outcomes**

To assess whether our results were partly driven by the patients’ antipsychotic medication, the CPZ equivalents (mg/d) of their medication were exploratorily correlated with the main behavioral and neural outcomes. For this purpose, the medication doses were converted into CPZ equivalents using the package “chlorpromazineR” for the software R^11^ version 4.2.2. The conversion factors were mainly derived from Gardner et al. (2010).^12^ For cariprazine and prothipendyl the conversion factors were derived from Leucht et al. (2016)^13^ and Leucht et al. (2020),^14^ respectively. The resulting CPZ equivalents were correlated with the levels of behavioral suppression (passive – active; derived from the detection thresholds) for each of the four experimental conditions (HandUni, HandBi, ToolUni, ToolBi). They were additionally correlated with the levels of neural suppression (passive – active; derived from the contrast estimates extracted as eigenvariates) derived from the two clusters of the four-way interaction contrast (see section 3.2.2 in the main manuscript) for each of the four experimental conditions. All correlations and the corresponding Bayes factors were calculated with JASP.

At the behavioral level (N = 42; mean CPZ equivalents = 504.511, *SD* = 416.704), none of the correlations between CPZ equivalents and behavioral suppression reached significance (HandUni: *r* = .033, *p* = .837, BF_10_ = .196; HandBi: *r* = .188, *p* = .233, BF_10_ = .383; ToolUni: *r* = .134, *p* = .397, BF_10_ = 272; ToolBi: *r* = -.022, *p* = .892, BF_10_ = .194).

Additionally, at the neural level (N = 20; mean CPZ equivalents = 448.615, *SD* = 480.115), correlations between CPZ equivalents and neural suppression obtained from the first cluster (peak in right cuneus; HandUni: *r* = -.157, *p* = .508, BF_10_ = .340; HandBi: *r* = -.122, *p* = .638, BF_10_ = .307; ToolUni: *r* = -.297, *p* = .203, BF_10_ = .590; ToolBi: *r* = -.066, *p* = .783, BF_10_ = .287) and the second cluster (peak in right MOG; HandUni: *r* = -.297, *p* = .203, BF_10_ = .591; HandBi: *r* = -.063, *p* = .792, BF_10_ = .286; ToolUni: *r* = -.064, *p* = .788, BF_10_ = .286; ToolBi: *r* = -.291, *p* = .213, BF_10_ = .573) of the interaction contrast were not significant.

These results indicate that the levels of suppression on the behavioral and neural level for patients were not influenced by their antipsychotic medication. Thus, the medication cannot explain the main results presented in this study.

**S9. Exploratory correlation analysis between symptom severity and the main behavioral and neural outcomes**

Similar as described in S8 we also performed exploratory correlation analyses between the scores of ego-disturbances as well as hallucinations of our patient sample and the levels of behavioral suppression (passive – active; derived from the percentage of “yes” responses) for each of the four experimental conditions. Additionally, the same scores were correlated with the levels of neural suppression (passive – active; derived from the contrast estimates extracted as eigenvariates) derived from the two clusters of the four-way interaction contrast. For hallucinations the SAPS subscore I was used and for ego-disturbances, the SAPS items 15-19 (see^15^).

At the behavioral level (N = 41; mean SAPS score for ego-disturbances = .312, *SD* = .585), none of the correlations between ego-disturbances and behavioral suppression reached significance (HandUni: *r* = .220, *p* = .166, BF_10_ = .491; HandBi: *r* = .113, *p* = .482, BF_10_ = .247; ToolUni: *r* = -.043, *p* = .788, BF_10_ = .201; ToolBi: *r* = -.054, *p* = .736, BF_10_ = .205). Similarly, correlations between hallucinations and behavioral suppression were not significant (N = 41; mean SAPS score for hallucinations = 2.049, *SD* = 4.111; HandUni: *r* = .156, *p* = .329, BF_10_ = .309; HandBi: *r* = .156, *p* = .330, BF_10_ = .308; ToolUni: *r* = -.121, *p* = .451, BF_10_ = .256; ToolBi: *r* = .037, *p* = .817, BF_10_ = .200).

At the neural level (N = 20; mean SAPS score for ego-disturbances = .240, *SD* = .370), correlations between ego-disturbances and neural suppression obtained from the first cluster (peak in right cuneus: HandUni: *r* = .081, *p* = .734, BF_10_ = .292; HandBi: *r* = .095, *p* = .689, BF_10_ = .298; ToolUni: *r* = -.164, *p* = .489, BF_10_ = .346; ToolBi: *r* = -.165, *p* = .487, BF_10_ = .347) and the second cluster (peak in right MOG; HandUni: *r* = .185, *p* = .436, BF_10_ = .368; HandBi: *r* = .209, *p* = .376, BF_10_ = .4; ToolUni: *r* = .252, *p* = .283, BF_10_ = .474; ToolBi: *r* = -.302, *p* = .195, BF_10_ = .607) of the interaction contrast were not significant. Similarly, correlations between hallucinations and neural suppression obtained from the same clusters did not reach significance (N = 20; mean SAPS score for hallucinations = 2.0, *SD* = 4.425; peak in right cuneus: HandUni: *r* = -.169, *p* = .476, BF_10_ = .351; HandBi: *r* = .08, *p* = .738, BF_10_ = .292; ToolUni: *r* = .162, *p* = .496, BF_10_ = .344; ToolBi: *r* = -.072, *p* = .761, BF_10_ = .289; peak in right MOG: HandUni: *r* = .28, *p* = .232, BF_10_ = .541; HandBi: *r* = .129, *p* = .587, BF_10_ = .318; ToolUni: *r* = .36, *p* = .119, BF_10_ = .861; ToolBi: *r* = -.061, *p* = .797, BF_10_ = .285).

To conclude, our data do not provide evidence for direct correlations between behavioral or neural suppression effects and the severity of hallucinations or ego-disturbances in our patient sample (see the limitations section in the main manuscript for further discussion).

**References**

1. Brickenkamp R. D2 Aufmerksamkeits-Belastungs-Test. 8th ed. Göttingen: Hogrefe; 1994.

2. Oldfield RC. The assessment and analysis of handedness: The Edinburgh inventory. Neuropsychologia. 1971;9(1):97-113. doi:10.1016/0028-3932(71)90067-4

3. Lehrl S, Triebig G, Fischer B. Multiple choice vocabulary test MWT as a valid and short test to estimate premorbid intelligence. Acta Neurologica Scandinavica. 1995;91(5):335-345. doi:10.1111/j.1600-0404.1995.tb07018.x

4. Andreasen NC. The Scale for the Assessment of Negative Symptoms (SANS). 1983;155:49-52.

5. Andreasen NC. The Scale for the Assessment of Positive Symptoms (SAPS). Published online 1984.

6. Army Individual Test Battery. Manual of directions and scoring. Published online 1944.

7. Petermann F. WAIS-IV. Wechsler Adult Intelligence Scale. Frankfurt: Pearson; 2012.

8. Pazen M, Uhlmann L, van Kemenade BM, Steinsträter O, Straube B, Kircher T. Predictive perception of self-generated movements: Commonalities and differences in the neural processing of tool and hand actions. NeuroImage. 2020;206:116309. doi:10.1016/j.neuroimage.2019.116309

9. Slotnick SD. Cluster success: fMRI inferences for spatial extent have acceptable false-positive rates. Cognitive Neuroscience. 2017;8(3):150-155. doi:10.1080/17588928.2017.1319350

10. Rolls ET, Huang CC, Lin CP, Feng J, Joliot M. Automated anatomical labelling atlas 3. NeuroImage. 2020;206:116189. doi:10.1016/j.neuroimage.2019.116189

11. R Core Team. R: A language and environment for statistical computing. R Foundation for Statistical Computing, Vienna, Austria. URL https://www.R-project.org/. Published online 2021.

12. Gardner DM, Murphy AL, O’Donnell H, Pharm B, Centorrino F, Baldessarini RJ. International Consensus Study of Antipsychotic Dosing. American Journal of Psychiatry. Published online 2010.

13. Leucht S, Samara M, Heres S, Davis JM. Dose Equivalents for Antipsychotic Drugs: The DDD Method. SCHBUL. 2016;42(suppl 1):S90-S94. doi:10.1093/schbul/sbv167

14. Leucht S, Crippa A, Siafis S, Patel MX, Orsini N, Davis JM. Dose-Response Meta-Analysis of Antipsychotic Drugs for Acute Schizophrenia. AJP. 2020;177(4):342-353. doi:10.1176/appi.ajp.2019.19010034

15. Roth MJ, Lindner A, Hesse K, Wildgruber D, Wong HY, Buehner MJ. Impaired perception of temporal contiguity between action and effect is associated with disorders of agency in schizophrenia. Proc Natl Acad Sci USA. 2023;120(21):e2214327120. doi:10.1073/pnas.2214327120
